# Plants with promising antileishmanial activity in Colombia: A systematic review and meta-analysis

**DOI:** 10.1016/j.parepi.2025.e00467

**Published:** 2025-12-01

**Authors:** Carlos Nieto-Clavijo, Liliana Morales, Guillermo Zambrano, Andrés Delgado-Aldana, Zayda-Lorena Corredor-Rozo, Eliana Patricia Calvo, Dario Tinjacá, Jacqueline Chaparro-Olaya

**Affiliations:** a Laboratorio de Parasitología Molecular, Vicerrectoría de Investigaciones, Universidad El Bosque, Bogotá, Colombia; b Bacterial Molecular Genetics Laboratory, Vicerrectoría de Investigaciones, Universidad El Bosque, Bogotá, Colombia; c Grupo de Virología, Vicerrectoría de Investigaciones, Universidad El Bosque, Bogotá, Colombia; d Applied Chemistry Research Group-INQA, Pharmaceutical Chemistry Program, Universidad El Bosque, Bogotá, Colombia

**Keywords:** *Leishmania*, Antileishmanial activity, Plant extracts, Drug discovery, Colombia, Ethnopharmacology, meta-analysis, IC_50_, Selectivity index

## Abstract

**Introduction:**

Leishmaniasis remains a global public health challenge. The exceptional biodiversity of Colombia includes numerous plants that constitute a promising resource for the development of new antileishmanial therapies.

**Aim of the study:**

To systematically assess the *in vitro* antileishmanial activity of Colombian plants, estimate pooled IC₅₀ values through meta-analysis, and identify plant-derived preparations with favorable selectivity indices (SI) as potential candidates for further investigation.

**Materials and methods:**

A systematic search (2000–April 2025) of PubMed, EMBASE, and LILACS identified *in vitro* studies reporting IC₅₀ values of Colombian plant-derived preparations against *Leishmania spp.* A random-effects model was used to estimate pooled IC₅₀ values. Risk of bias was assessed using a modified QUIN tool. Subgroup analyses explored methodological and biological factors such as plant part, extraction solvent, and taxonomic family.

**Results:**

Thirteen studies provided complete data for meta-analysis. The pooled mean IC₅₀ was 37.89 μg/mL (95 % CI: 34.99–40.78), with substantial heterogeneity (I^2^ = 100 %), consistent with marked differences in plant species, plant parts, extraction solvents, *Leishmania* species/strains, cell lines, and assay protocols. Leaf essential oils of *Xylopia discreta*, crude leaf extracts of *Lippia origanoides* and *Moquilea salicifolia*, and bark/wood fractions of *Handroanthus chrysanthus*, exhibited potent *in vitro* leishmanicidal activity (IC₅₀ = 0.39–11.1 μg/mL) and high selectivity indices (SI = 18.9–134).

**Conclusion:**

Colombian plants represent a promising source of leishmanicidal agents, with several species exhibiting strong *in vitro* antileishmanial activity and remarkably high SI values. However, since SI thresholds were originally established for purified compounds, their interpretation for other plant-derived preparations should be approached with caution. Moving toward standardized assays and reporting practices will be key to translating these findings into reproducible and actionable knowledge.

## Highlights

• Meta-analysis of Colombian plant-derived preparations yielded a pooled mean IC₅₀ of 37.9 μg/mL.
• *Xylopia discreta* leaf oils showed potent, highly selective activity against L. *panamensis*.
• *Lippia origanoides* leaf extracts displayed the highest selectivity against L. *braziliensis*.
• Methodological variability strongly influenced reported bioactivity outcomes.
• Colombian plant-derived preparations show promising activity against clinically relevant *Leishmania* species.

## 1 Introduction

Leishmaniasis is a neglected tropical disease caused by protozoan parasites of the genus *Leishmania*, transmitted to humans through the bite of infected female *Phlebotomine* sandflies (*Phlebotomus spp.* in the Old World and *Lutzomyia spp.* in the New World) (World Health Organization, 2023). This vector-borne disease presents a wide clinical spectrum, with three main manifestations: cutaneous leishmaniasis (CL), the most prevalent form, characterized by ulcerative skin lesions that may result in disfiguring scars; mucocutaneous leishmaniasis (MCL), a severe and potentially mutilating progression of CL that affects the mucous membranes of the nasopharyngeal region; and visceral leishmaniasis (VL), the most severe form, which involves internal organs such as the spleen, liver, and bone marrow, leading to systemic complications and high mortality if left untreated (Torres-Guerrero et al., 2017; Alvar et al., 2012).

In Colombia, nine *Leishmania* species have been historically reported: *L. panamensis*, *L. braziliensis*, *L. guyanensis*, *L. equatoriensis*, *L. lainsoni*, *L. colombiensis*, *L. mexicana*, *L. amazonensis*, and *L*. *infantum*. All except *L*. *infantum*, which is associated with VL, have been linked to CL. Furthermore, *L. panamensis*, *L. braziliensis*, and *L*. *guyanensis* have also been associated with MCL (Ovalle-Bracho et al., 2019). In addition, *L*. *naiffi* and *L. lindenbergi* were identified more recently in three Colombian soldiers, representing the first reports of these species in the country (Correa-Cárdenas et al., 2020). Compared to 2023, an overall increase of 25.5 % in reported cases of leishmaniasis in Colombia was observed in 2024, from 5486 to 6886 notifications (Instituto Nacional de Salud, 2024; Instituto Nacional de Salud, 2025). This increase was mainly driven by a 26.2 % increase in CL, which continues to represent the most frequent clinical manifestation. In addition, a moderate increase of 6.6 % was observed in cases of MCL, while cases of VL decreased by 15 % (Instituto Nacional de Salud, 2025). The persistent epidemiological burden of leishmaniasis in Colombia, driven by its endemicity, high incidence rates, broad geographical distribution, and the diversity of *Leishmania* species, highlights the urgent need for improved disease management.

Conventional treatment strategies for leishmaniasis in Colombia rely primarily on the use of pentavalent antimonials, amphotericin B, miltefosine, and other pharmacological agents (Ministerio de Salud y Protección Social, 2023). Despite their widespread use, these therapies are associated with significant limitations, including severe toxicity, high cost, complex administration protocols, and, most critically, the increasing emergence of drug-resistant *Leishmania* strains. A recent systematic review addressing treatment failure and clinical relapse in leishmaniasis identified Latin America as one of the regions with the highest number of cases, with Brazil, Colombia, and French Guiana reporting the highest rates of relapse (Santos et al., 2023). The study also highlighted the impact of treatment failure across clinical forms of the disease, reporting failure or relapse rates of 47.6 % for CL and 45.2 % for VL. These findings underscore the urgent need for novel therapeutic strategies capable of overcoming the challenges posed by drug-resistant *Leishmania* strains.

Within the repertoire of pharmacological approaches, increasing attention is being paid to the reuse of existing drugs and the identification of new compounds with anti-leishmanial activity (Oualha et al., 2024; Scheiffer et al., 2024). In this context, natural products have emerged as a particularly promising area of exploration (Koko et al., 2022; Majumder et al., 2023). Plant-derived compounds offer a rich source of bioactive molecules that may lead to the development of safer, more effective, and affordable therapeutic options, addressing key gaps in current pharmacological interventions (Hassan et al., 2022).

Colombia's exceptional biodiversity presents a valuable opportunity for bioprospecting efforts. As one of the most biodiverse countries in the world, Colombia hosts a wide range of plant species, many of which produce compounds with antiparasitic activity (Pájaro-González et al., 2022). This phytochemical diversity constitutes a largely untapped resource for the discovery of new leishmanicidal compounds. Recent pharmacological and ethnobotanical studies have identified several species with activity against *Leishmania spp.* (Hassan et al., 2022), further supporting the need for a systematic evaluation of the available evidence.

This systematic review and meta-analysis aims to comprehensively evaluate research conducted between January 1, 2000, and April 30, 2025, on Colombian plants with documented antileishmanial activity. By synthesizing the available evidence, the study aims to identify candidate species with potential for the development of safer and more effective treatments for leishmaniasis. Additionally, it aims to identify current knowledge gaps and propose future research priorities to support the advancement of these natural products toward clinical application.

## 2 Methodology

### 2.1 Research question

Which Colombian plants have demonstrated *in vitro* antileishmanial activity based on reported IC₅₀ values?

### 2.2 Reporting system and registration

This systematic review and meta-analysis was conducted in accordance with the Preferred Reporting Items for Systematic Reviews and Meta-Analyses (PRISMA) (Page et al., 2021). The study protocol was prospectively registered in the International Prospective Register of Systematic Reviews (PROSPERO) under the registration number CRD420251076470, accessible at https://www.crd.york.ac.uk/PROSPERO/view/CRD420251076470.

### 2.3 Data sources

A comprehensive literature search was conducted in the PubMed (https://pubmed.ncbi.nlm.nih.gov/), EMBASE (https://www.embase.com/search/), and LILACS (https://lilacs.bvsalud.org/) databases. These platforms were selected for their complementary coverage of biomedical, pharmacological, and regional scientific literature. The search was restricted to articles published between January 1, 2000, and April 30, 2025.

### 2.4 Search strategy

The following MeSH descriptors served as the foundation of the search strategy in PubMed/MEDLINE: “Bioprospecting”, “Ethnopharmacology”, “Medicine, Traditional”, “Phytochemicals”, “Phytotherapy”, “Plant Extracts”, “Plants”, “Medicinal, Plant Oils”, “Oils, Volatile”, “Colombia”, “*Leishmania*”, “*Leishmania braziliensis*”, “*Leishmania donovani*”, “*Leishmania guyanensis*”, “*Leishmania infantum*”, “*Leishmania major*”, “*Leishmania mexicana*”, “*Leishmania tropica*”, and “Leishmaniasis”. All associated entry terms were included in the search strategy to ensure exhaustive retrieval. However, for “Leishmaniasis,” “*Leishmania,*” and all listed *Leishmania* species, preliminary testing showed that entry terms did not yield additional results beyond those retrieved by the main descriptors. To avoid unnecessary complexity, these entry terms were omitted from the final search string (S1 File). In Embase, each Emtree term was exploded using the /exp. function to capture all narrower terms within the hierarchical structure. In addition, all synonyms were included for each concept. This procedure was applied to all terms except Colombia and Colombian, as their listed synonyms, Columbia and Colombians, are incorrect: the former refers to a different geographic entity, and the latter is not a valid term (S1 File). In LILACS, the search was conducted using official DeCS (“Descriptores en Ciencias de la Salud,” meaning “Health Sciences Descriptors”), a multilingual controlled vocabulary created by BIREME/PAHO/WHO. The search terms were applied in English, Spanish, and Portuguese. Because many records in LILACS are published in Spanish or Portuguese without English abstracts, inclusion of all three languages was essential to capture regionally relevant literature. Due to character limits in the LILACS search interface, the final search string included only the official DeCS descriptors, omitting their extensive lists of synonyms. However, an initial exhaustive search (incorporating all DeCS synonyms) was performed, confirming that their inclusion did not alter the retrieval output. Thus, the compacted version preserved the same sensitivity and recall as the fully expanded strategy (S1 File). An information specialist assisted in developing and refining the search terms. The search covered articles published between January 1, 2000, and April 30, 2025, with no language restrictions. Reporting followed the PRISMA 2020 checklist (S1 Checklist).

### 2.5 Eligibility criteria

Studies were included if they (i) were original research articles; (ii) evaluated the *in vitro* leishmanicidal activity of plant-derived preparations obtained from species collected in Colombia; and (iii) reported IC₅₀ or EC₅₀ values for antiparasitic activity. Studies were excluded if they: (i) did not report IC₅₀/EC₅₀ values; (ii) did not clearly confirm the Colombian origin of the plant material or failed to cite a primary source verifying it; (iii) were not available in full text; (iv) were publication types lacking complete primary data (*e.g.*, conference abstracts, reviews, letters, posters, infographics); (v) relied exclusively on *in silico* methods; or (vi) evaluated only synthetic molecules rather than plant-derived preparations.

### 2.6 Study selection and quality assessment

Study selection was performed independently by three reviewers (CNC, LM, JCO) using Rayyan (Ouzzani et al., 2016). After importing all retrieved records, duplicates were removed, and titles and abstracts were screened independently. Any discrepancies were resolved by consensus, after which full texts were assessed for eligibility based on the predefined criteria.

Data extraction was performed using a standardized template in Microsoft Excel. Five reviewers (CNC, LM, JCO, ADA, GZ) independently extracted the following variables: bibliographic details; botanical information (family, species, plant part); extraction and fractionation procedures; *Leishmania* species, parasite stage and strain; assay parameters (concentration range, parasite inoculum, culture medium, incubation time, parasite count method), control drugs used in the antileishmanial assays, IC₅₀/EC₅₀ values, standard deviation, selectivity index; cell line used for cytotoxicity assessment; and number of experimental replicates. The compiled dataset was subsequently reviewed and cross-validated by CN and JCO to ensure accuracy and consistency. Only studies reporting IC₅₀ or EC₅₀ values were included. No values were estimated from graphical plots, and study authors were not contacted to obtain missing information. This strategy minimized availability bias, ensured uniformity across extracted outcomes, and enhanced reproducibility.

Methodological quality and risk of bias were assessed using an adapted version of the Quality Assessment Tool for *In Vitro* Studies (QUIN) (Sheth et al., 2024). Four reviewers (CNC, LM, JCO, ADA) independently evaluated each study. Items were scored as follows: 2 points if clearly reported, 1 if insufficiently specified, and 0 if not reported. Scores were converted to percentages and studies classified as poor (<50 %), moderate (50–75 %), or high (>75 %) quality. All scores and supporting notes were documented in a standardized Excel file, and discrepancies were resolved through discussion until consensus was reached.

### 2.7 Statistical analysis

To estimate the pooled inhibitory effect of Colombian plant-derived preparations against *Leishmania spp.*, a meta-analysis was conducted by calculating the mean IC₅₀ values and their corresponding 95 % confidence intervals (CIs) from the included studies. Given the anticipated heterogeneity across studies, a random-effects model was applied. Forest plots were generated to visualize both individual and pooled IC₅₀ estimates along with their CIs. Between-study heterogeneity was assessed using Cochran's Q test (with *p* < 0.1 indicating significant heterogeneity) and the *I*^*2*^ statistic, interpreted as low (25 %–49 %), moderate (50 %–74 %), or high (≥75 %). Potential publication bias was evaluated using funnel plots and Egger's test. All statistical analyses were performed using Stata 16.0 software (StataCorp, College Station, TX, USA).

## 3 Results

### 3.1 Study selection and data classification

**Fig. 1:**
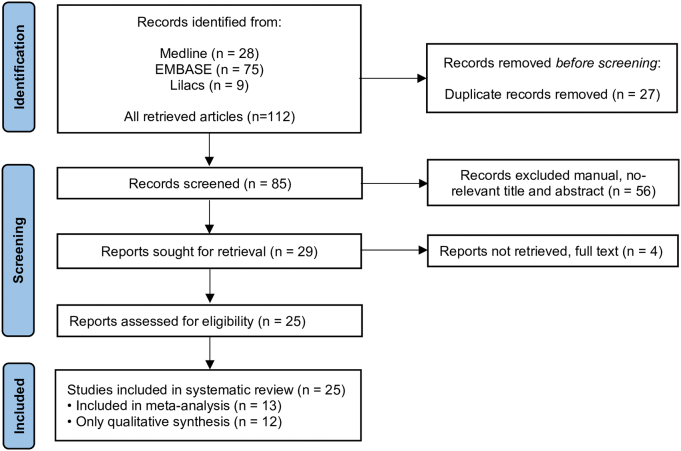
PRISMA flow diagram illustrating the search and selection process.

A total of 112 articles were initially retrieved from the database searches. After removing 27 duplicates, the titles and abstracts of 85 studies were screened. Of these, 56 were excluded because they did not meet the eligibility criteria, mainly due to being non–peer-reviewed sources, involving non-Colombian plant species, addressing unrelated or non-experimental topics (*e.g.*, reviews, ethnobotanical, epidemiological, or *in silico* studies), or lacking data on antileishmanial activity (S1 Table). The remaining 29 articles were assessed in full text. Subsequently, four additional studies were excluded due to incomplete data, the use of non-Colombian plant material, or the inclusion of non–plant-derived data (S1 Table). Ultimately, 25 studies met all inclusion criteria and were retained for analyses (Fig. 1, S2 Table). Of these, 13 provided IC₅₀ values together with measures of variability (*e.g.*, standard deviations and number of replicates) and were therefore included in the meta-analysis (S2 Table).

### 3.2 Risk of bias assessment

**Fig. 2:**
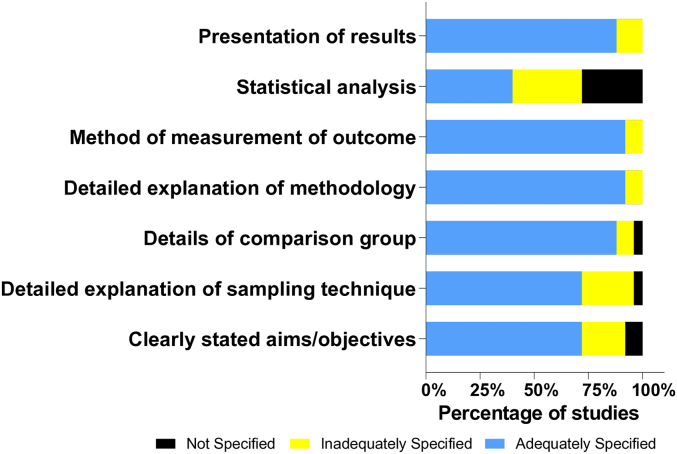
Methodological quality across six key domains in the included studies.

To assess methodological quality and reporting standards, the included studies were evaluated using a modified QUIN tool for *in vitro* research (S3 Table). The results are summarized in Fig. 2. Most of the included studies demonstrated adequate methodological clarity across most assessed domains, particularly in the presentation of results, outcome measurement methods, and the overall description of the experimental procedures. However, the domain with the most frequent shortcomings was statistical analysis. A substantial proportion of studies (about 20 %) lacked sufficient detail, and nearly 30 % did not specify the statistical procedures at all. This indicates that, although quantitative data were available, many studies provided limited detail on how variability, statistical significance, or effect sizes were assessed, potentially compromising the reproducibility and comparability of their findings. Additional methodological limitations were noted in the reporting of sampling techniques, and the clarity of study aims or objectives. These elements were inadequately described or omitted in 25 % and 20 % of the studies, respectively. Incomplete reporting of sampling strategies is particularly relevant in plant-based studies, where ecological variability and harvesting protocols can influence phytochemical content and, consequently, bioactivity outcomes. In contrast, over 90 % of the studies clearly described the comparison groups and methodological procedures, suggesting a generally robust experimental design and implementation, despite shortcomings in other reporting areas.

These findings highlight the need for greater adherence to standardized reporting guidelines in *in vitro* pharmacological research, to improve clarity, reproducibility, and quality assessment of preclinical evidence derived from natural products.

The y-axis lists the evaluated methodological domains. The x-axis represents the percentage of studies assessed. Bars are stacked and divided into three colors indicating the level of reporting: Blue = adequately reported, yellow = partially reported, and black = not reported. Each bar sums to 100 % and shows the proportion of studies falling into each reporting category per domain.

### 3.3 Characteristics of plant-derived preparations

From the 13 studies included in the meta-analysis, 120 IC₅₀ records were extracted, corresponding to crude extracts, essential oils, fractions, or isolated compounds. For simplicity, when referring collectively to these different preparation types, we use the term “plant-derived preparations.” However, individual samples are always described using their specific preparation type. These records corresponded to preparations derived from 24 plant species belonging to 18 botanical families, all collected in Colombia and evaluated *in vitro* against different *Leishmania* species (Table 1).

**Table 1:** Summary of plant species, plant material preparation, and reported IC₅₀ values in studies included in the meta-analysis.

Ref.	Plant			Plant material preparation				*Leishmania*			Antileishmanial assay		
	Family	Specie	Part	Extraction technique	Extract	Fractions technique	Fraction	Sp	St	Strain	Cell	IC50 (μg/mL)±SD	SI
Cardona-G et al., 2020	Araliaceae	*Oreopanax incisus*	Leaf	Percolation. Solvent	Dichloromethane	NA	NA	Lp	IA	M/HOM/CO/87/UA-140-epir-GFP	U937	24.6±1.1*	2

					Ethyl acetate							23.7±1*	2.2

	Hexane	87.4±7.6*			>2.30						

	Chrysobalanaceae	*Moquilea salicifolia*			Ethyl acetate							9.8±1.2*	**>20.40**

	Lauraceae	*Persea ferruginea*			Dichloromethane							48±7.3*	>4.20

	Ethyl acetate	25.5±2.6*	>7.80								

Rubiaceae	*Palicourea winkleri*	Dichloromethane	21.5±2.79*	3.57

		Ethanol	29.4±0.5*	>6.81

Ethyl acetate	14.1±0.47*	7.75

Hexane	84.8±2.5*	0.14

Control drug 1: Meglumine antimoniate							6.3±0.9*	78.6

Control drug 2: Amphotericin B							0.04±0.01*	1052.5

Cervantes-Ceballos et al., 2023	Malvaceae	*Malachra alceifolia*	Leaf	Maceration. Solvent	Ethanol	NA	NA	Lmx	AA	MHOM/VE/60/Ltrod	RAW 264.7	15.65±0.74*	2.67

	Column chromatography. Silica gel	Chloroform fraction	5.78±0.46*	8.48							

Control drug : Miltefosine							Toxicity data not shown	

Chávez-Enciso et al., 2014	Lauraceae	*Ocotea macrophylla*	Leaf	Maceration. Solvent	Ethanol	NA	NA	Lm	IA	Friedlin V1	PMH	29.16±2.08*	3.45

								P	9.7±1*	**10.36**	

	Lp	IA	M/HOM/CO/87/UA-140			12.67±0.11*	7.93				

	P	100.64±2.96*	1								

	Rutaceae	*Zanthoxylum schreberi*	Bark/Wood			Successive CHCl₃ / CHCl₃–EtOH (80:20) partition	Alkaloidal fraction	Lm	IA	Friedlin V1		30.135±0.685*	**10.5**

								P	77.04±3.72*	4.11	

				Lp	IA	M/HOM/CO/87/UA-140	46.23±1.05*	6.85			

				P	61.43±3.05*	5.15					

NA	NA	Lm	IA	Friedlin V1	24.95±3.15*	2.86

		P	28.99±3.17*	2.46

Lp	IA	M/HOM/CO/87/UA-140	6.16±0.115*	**11.6**

P	17.06±1495*	4.19

	Control drug 1: Pentamidine							Lm	Friedlin V1	0.31±0.061	20

Lp	M/HOM/CO/87/UA-140	0.17±0.003	35

Control drug 2: Sodium stibogluconate							Lm	IA	Friedlin V1	932.8±289.63	3

Lp	M/HOM/CO/87/UA-140	704.7±361.6	4

Correa et al., 2025	Bignoniaceae	*Handroanthus chrysanthus*	Bark/Wood	Percolation. Solvent	Ethanol	NA	NA	Lb	IA	M/HOM/CO/88/UA-301-EGFP	U937	17.7±1.4*	3.4

						Exclusion chromatography on Sephadex LH-20	Methanolic fraction 1					7.9±0.3*	1.7

							Methanolic fraction 2					11.1±1.4*	**18.9**

							Methanolic fraction 3					6.7±1.7*	1.2

	Methanolic fraction 4	3.3±0.3*	2.4								

Methanolic fraction 5	23.3±4.2*	<2.57

Successive chromatography on silica gel from Fraction	Sativan. Compound from fraction 2	5.5 ± 0.5	1.3

Vestitol. Compound from fraction 2	5.3 ± 0.4	1.9

Control drug : Amphotericin B							0.3 ± 0.1	122

Correa et al., 2014	Sapindaceae	*Sapindus saponaria*	Fruit	Maceration. Solvent	Ethanol	NA	NA	Lp	IA	M/HOM/CO/87/UA-140 (GFP)	U937	14.5±0.8*	1.8

								Lp	AA	M/HOM/CO/87/UA-140		57.7±0.6*	0.5

								La	P	IFLA/BR/75/PH8		23.4±0.007	NR

						Lb	M/HOM/BR/75/M2903	32±0.14		NR	

						Ld	M/HOM/BR/74/PP75	35.35±3.88		NR	

						Column chromatography on Sephadex LH-20 with Hexane-Chloroform-Methanol (2:1:1)	Hederagenin-3-O-(3.4-O-diacetyl-ß-D-xylopiranosyl-(1→3)-α-L-rhamnopyranosyl-(1→2)-α-L-arabinopyranoside.	La	IFLA/BR/75/PH8	23±0.56		NR

								Lb	M/HOM/BR/75/M2903	32±0.21		NR

								Ld	M/HOM/BR/74/PP75	68.3±0.49		NR

							Lp	IA	M/HOM/CO/87/UA-140 (GFP)	2.5±0.3*		0.5

							Lp	AA	M/HOM/CO/87/UA-140	8.6±2.3*		0.2

	Hederagenin-3-O-(3.4-O-diacetyl-α-L-arabinopyranosyl-(1→3)-α-L-rhamnopyranosyl-(1→2)-α-L-arabinopyranoside	La	P	IFLA/BR/75/PH8	68.4±0.35		NR				

Lb		M/HOM/BR/75/M2903		32.4±0.28	NR	

Ld		M/HOM/BR/74/PP75	68.6±0.14	NR		

Lp	IA	M/HOM/CO/87/UA-140 (GFP)	2.7±0.6*	0.8

Lp	AA	M/HOM/CO/87/UA-140	16.6±0.1*	0.1

Hederagenin-3-O-(4-O-diacetyl-ß-D-xylopiranosyl-(1→3)-α-L-rhamnopyranosyl-(1→2)-α-Larabinopyranoside	La	P	IFLA/BR/75/PH8	23.2±0.28	NR

	Lb		M/HOM/BR/75/M2903	28.1±0.42	NR

	Ld	M/HOM/BR/74/PP75	66.3±1.34	NR

Lp	IA	M/HOM/CO/87/UA-140 (GFP)	2.1±0.2*	1.8

Lp	AA	M/HOM/CO/87/UA-140	10.8±1.1*	0.3

Control drug 1: Amphotericin							NR	P	NR	0.2	NR

Control drug 2: Pentamidine							10	NR

López et al., 2009	Annonaceae	*Xylopia discreta*	Leaf	Hydrolysis	Aqueous	NA	NA	Lp	IA	M/HOM/CO/87/UA-140	J774	9.68±5*	**32.6**

				Maceration. Solvent	Ethanol						U937	25±9.7*	**>40.00**

					J774						48.7±12*	8

					Ether						10.6±7.3*	7.4

					U937						50±13*	3.82

					Ethyl acetate	J774	30.2±8.9*				7.4

			U937		50±19*	2.32				

	Methanol	37.5±14*	**22.87**							

J774	9.23±1.2*	**64.8**

Ethanol	Hydrodistillation	Essential oil	U937	6.25±1.1*	**25.6**

Essential oil	J774	6.35±4*	**110**

Seed		Chloroform	NA	NA	46.1±6*	4.7

	Ethanol	3.68±0.98*			**11.6**

U937	6.25±2.6*	5.66

Control drug : Pentamidine							J774	0.59±0.23*	260

U937	1.12±.1.7*	197

Martínez et al., 2010	Solanaceae	*Physalis peruviana*	Flower	Percolation. Solvent	Ethanol	NA	NA	Lp	AI	M/HOM/CO/87/UA-140 (GFP)	J774	8.9±1*	1.7

						Liquid–liquid partitioning. Methanol/water	Water-insoluble fraction					28±5*	1.25

	Water-soluble fraction	30±6.2*	1.04								

Ether	Polar fraction	102±33*	0.53

Control drug : Amphotericin B							0.08±0.03*	271.25

Neira et al., 2018	Verbenaceae	*Lippia alba*	Leaf	Microwave-assisted hydrodistillation	Aqueous - Essential oil chemotype citral	NA	NA	Lb	IA	M/HOM/BR/75/M2903	THP-1	9.19±2.1*	2.8

								P		8.88±1.84*		2.9

		Lp			IA			M/HOM/PA/71/LS94	63.87±6.2*	0.4	

		*Lippia origanoides*			Aqueous - Essential oil chemotype phellandrene			Lb	P	M/HOM/BR/75/M2903		11.18±1.63*	6.8

					Aqueous - Essential oil chemotype thymol				0.39±0.079*			**134.7**

	IA		15.43±0.9*	3.4							

Lp		M/HOM/PA/71/LS94	42.66±2*	1.2

Aqueous - Essential oil chemotype carvacrol (AE3)	89.29±4.2*	0.5

Lb	P	M/HOM/BR/75/M2903	0.57±0.037*	**76.7**

Control drug : Amphotericin B							Lp	IA	M/HOM/PA/71/LS94	0.49±40.18*	32

Lb	M/HOM/BR/75/M2903	0.08±0.1*	196

Pérez et al., 2016	Aquifoliaceae	*Ilex laurina*	Leaf	Percolation. Solvent	Dichloromethane	NA	NA	Lp	AA	M/HOM/CO/87/UA-140-epir-GFP	U937	12.3±0.8*	1.4

					IA				20.3±3.2*			0.8

					Ethyl acetate	AA	52.8±1.6*		1.1		

	IA	7.5±1.5*	7.7								

	Thin-Layer Chromatography	Dichloromethane-methanol-acetic acid. isolation Ilexsaponin A.	IA	5.9±0.5*	4.3						

Dichloromethane-methanol-acetic acid. isolation Rotungenoside	AA	41.6±1.5*	0.6

Control drug 1: Amphotericin B							AA	0.06±0.01*	443.3

IA	0.04±0.01*	532

Control drug 2: Sb(V) meglumine antimoniate							IA	6.3±0.9*	78.7

Robledo et al., 2015	Picramniaceae	*Picramnia gracilis*	Fruit	Percolation. Solvent	Ethanol	NA	NA	Lp	IA	M/HOM/CO/87/UA-140-epir-GFP	U937	35.7±1.3*	>2

	Silica gel column chromatography	5.3´-Hydroxy-7.4´-dimethoxyflavanone	17±2.8*	**>11.8**							

Control drug : Amphotericin B							37.5±7.6*	625

Sánchez-Suárez et al., 2011	Lauraceae	*Ocotea macrophylla*	Leaf	Maceration. Solvent	Ethanol	NA	NA	Lp	P	M/HOM/CO/87/UA-140	J774	98±19.9*	2

						Silica gel column chromatography	Erythro-diarylbutane					26.6±2.6*	3

							Ocophyllal-A	58.9±32.2*		3	

		Ocophyllal-B				Lb	M/HOM/CO/86/CL-250	36.3±11.4*		4	

		Lp				M/HOM/CO/87/UA-140	34±14.9*	4			

		NA				NA	Lb	M/HOM/CO/86/CL-250		85.7±22.9*		2

	*Pleurothyrium cinereum*	Silica gel column chromatography	(+)-otobaphenol	Lp	M/HOM/CO/87/UA-140	31.1±13.9*	6				

7,8,7’,8’-didehydrootobaphenol			55.6±5.4*	3

Dihydroflavokawin-B			Lb	M/HOM/CO/86/CL-250	25±15*	2

Lp	M/HOM/CO/87/UA-140	19.9±3.3*	2

Kaurenoic acid	Lb	M/HOM/CO/86/CL-250	45.4±4.8*	1

Lp	M/HOM/CO/87/UA-140	25.3±9.2*	1

Control drug : Pentamidine isethionate							Lp	M/HOM/CO/87/UA-140	0.5±0.1*	16

Lb	M/HOM/CO/86/CL-250	0.6±0.2*	12

Sánchez-Suárez et al., 2013	Apiaceae	*Coriandrum sativum*	Seed	Essential oils obtained by hydrodestillation	Aqueous	NA	NA	Lp	P	M/HOM/CO/98/UA-1702	J774	427.95±118.4*	3

	Lamiaceae	*Ocimum basilicum*	Leaf					Lg		M/HOM/CO/84/CL-007		315.55±90.86*	>5.00

		Lp						M/HOM/CO/98/UA-1702		251.59±64.18*		>6.40

		*Origanum vulgare*						Lb		M/HOM/CO/2011/UA-3320		204.36±21.56*	2.7

	Lm							Friedlin V1		171.8±20.64*		3.2

	Lp	M/HOM/CO/98/UA-1702						42.23±2.04*		**12.9**	

	*Thymus vulgaris*	M/HOM/CO/98/UA-1702						402.23±82.9*		1.1	

	Poaceae	*Cymbopogon citratus*						Lb		M/HOM/CO/2011/UA-3320		160.06±43.49*	1.3

				Lg	M/HOM/CO/84/CL-007	149.1±6.22*	1.4				

		Lm	Friedlin V1	194.05±29.2*	1.1						

Lp	M/HOM/CO/98/UA-1702	180.83±82.24*	1.2

*Citrus × limon*	Lg	M/HOM/CO/84/CL-007	231.4±42.43*	>6.90

Zingiberaceae	*Zingiber officinale*	Lb	M/HOM/CO/2011/UA-3320	124.94±52.98*	1.2

		Lg	M/HOM/CO/84/CL-007	256.95±75.17*	0.6

Lm	Friedlin V1	303±107.48*	0.5

Lp	M/HOM/CO/98/UA-1702	154.83±23.86*	1

Control drug: Pentamidine							Lp	M/HOM/CO/98/UA-1702	0.049±0.004*	94.7

							Lb	M/HOM/CO/2011/UA-3320	0.65±0.28*	7.1

Lm	Friedlin V1	0.24±0.004*	19.3

Lg	M/HOM/CO/84/CL-007	0.06±0.002*	77.3

Torres et al., 2020	Fabaceae	*Cabari brunnea*	Bark/Wood	Maceration. Solvent	Ethanol	NA	NA	Lb	IA	M/HOM/CO/88/UA-301-EGFP	U937	15.4±2.7*	0.7

						Sephadex LH-20 column chromatography	F1-Methanolic					88.4±25*	2.6

							F3-Methanolic. Isolation 3.5-diOMe stilbene					4.2±0.21*	<1.40

F3-Methanolic. Isolation Pinostrobin	13.6±0.28*	3.6

F4-Methanolic	145.9±48.2*	2.8

Control drug : Amphotericin B							0.3 ± 0.1*	122

IC₅₀ refers to the concentration required to inhibit 50% of parasite viability. In cases marked with an asterisk (*), inhibition was determined using EC₅₀. The selectivity index (SI), calculated as the ratio of cytotoxic concentration to effective antiparasitic concentration, provides an estimate of therapeutic potential. SI values greater than ten (10) are shown in bold, as this threshold is commonly considered a minimum criterion for preclinical evaluation, indicating reduced toxicity to mammalian cells. Abbreviations: **NA;** not applicable. **NR;** not reported. **Sp;** species. **St;** stage. **Ld;***L. donovani*. **La;***L. amazonensis*. **Lb;***L. braziliensis*. **Lp;***L. panamensis*. **Lg;***L. guyanensis*. **Lm;***L. major.***Lmx;***L. mexicana*. **IA;** intracellular amastigote. **AA;** axenic amastigote. **P;** promastigote. The column “**Cell**” indicates the mammalian cell lines used either for *Leishmania* infection assays or for cytotoxicity testing to calculate the SI, both for promastigotes and for axenic or intracellular amastigotes. The full dataset from all antileishmanial assays is provided in the Supplementary data (S5 Table).

The most frequently tested parasite was *L*. *panamensis* (*n* = 67), followed by *L*. *braziliensis* (*n* = 30) (Table 2). The promastigote stage was the most evaluated form (*n* = 68) (Table 1). Regarding plant taxonomy, the most represented families were Sapindaceae (*n* = 20), Lauraceae (*n* = 18), and Annonaceae (*n* = 14), followed by Verbenaceae (*n* = 9), Bignoniaceae and Rutaceae (*n* = 8 each), Aquifoliaceae and Lamiaceae (n = 6 each), and Fabaceae and Poaceae (*n* = 5 each). Less represented families included Rubiaceae, Solanaceae, and Zingiberaceae (*n* = 4 each), Araliaceae (n = 3), Malvaceae and Picramniaceae (n = 2 each), and Apiaceae and Chrysobalanaceae (n = 1 each).

**Table 2:** Subgroup analyses of antileishmanial activity by type of preparation, plant part, extraction solvent, *Leishmania* species, and botanical species.

Categories	n	IC_50_ (μg/mL)	95 % CI		Heterogeneity	
			Lower	Upper	*I*^2^ (%)	*p value*
Type of preparation						
Extracts	71	43.30	39.69	46.91	100,00	0.000
Fractions/Compounds	49	30.90	23.83	37.96	100,00	0.000

Part of the plant						
Bark wood	21	22.39	19.61	25.17	99.90	0.000
Flower	4	36.99	20.74	53.24	99.00	0.000
Fruit	22	30.42	23.70	37.14	100,00	0.000
Leaf	69	39.28	36.83	41.72	99.90	0.000
Seed	4	24.99	10,00	39.97	99.40	0.000

Solvent						
Aqueous	26	38.72	36.37	41.08	99.70	0.000
Chloroform**	1**	46.10	42.18	50.02	**NA**	**NA**
Dichloromethane	5	24.86	17.32	32.41	99.40	0.000
Ethanol	71	29.20	25.71	32.68	100,00	0.000
Ether	3	52.79	12.58	93.01	98.30	0.000
Ethyl acetate	10	25.59	17.63	33.55	99.90	0.000
Hexane**	2**	85.05	83.15	86.95	0.00	0.426
Methanol**	2**	22.98	4.71	50.67	97.30	0.000

*Leishmania* specie						
*L. amazonensis*	4	34.5	16.34	52.65	99.90	0.000
*L. braziliensis*	30	29.34	23.49	35.20	99.60	0.000
*L. donovani*	4	61.03	58.06	64,00	99.60	0.000
*L. guyanensis*	4	230.48	156.04	304.91	100,00	0.000
*L. major*	9	63,00	50.68	75.32	100,00	0.000
L. mexicana**	2**	10.70	1.03	20.37	91.60	0.000
*L. panamensis*	67	32.51	29.90	35.04	99.30	0.000

Plant species (family)						
*Cabari brunnea* (Fabaceae)	5	22.12	14.95	29.29	99.90	0.000
*Citrus × limon* (Poaceae)**	1**	231.4	189.81	272.98	**NA**	**NA**
*Coriandrum sativum* (Apiaceae)**	1**	427.95	311.88	544.01	**NA**	**NA**
*Cymbopogon citratus* (Poaceae)	4	167.31	139.57	195.04	69.20	0.021
*Handroanthus chrysanthus* (Bignoniaceae)	8	9.36	7.20	11.52	99.00	0.000
*Ilex laurina* (Aquifoliaceae)	6	23.39	9.85	36.93	99.90	0.000
*Lippia alba* (Verbenaceae)	3	26.51	8.34	44.68	98.70	0.000
*Lippia origanoides* (Verbenaceae)	6	17.71	15.79	19.63	99.80	0.000
*Malachra alceifolia* (Malvaceae)**	2**	10.7	1.03	20.37	99.60	0.000
*Moquilea salicifolia* (Chrysobalanaceae)**	1**	9.8	8.84	10.76	**NA**	**NA**
*Ocimum basilicum* (Lamiaceae) **	2**	275.48	214.84	336.13	24.40	0.250
*Ocotea macrophylla* (Lauraceae)	10	47.27	35.52	59.02	99.70	0.000
*Oreopanax incisus* (Araliaceae)	3	43.58	33.65	53.50	99.50	0.000
*Origanum vulgare* (Lamiaceae)	3	139.12	22.42	255.82	99.50	0.000
*Palicourea winkleri (*Rubiaceae*)*	4	37.42	22.15	52.68	100,00	0.000
*Persea ferruginea* (Lauraceae)**	2**	36.57	14.53	58.62	98.00	0.000
*Physalis peruviana* (Solanaceae)	4	36.99	20.74	53.24	99.00	0.000
*Picramnia gracilis* (Picramniacea)**	2**	26.37	8.05	44.70	99.50	0.000
*Pleurothyrium cinereum* (Lauracea)	6	33.91	19.51	48.32	98.00	0.000
*Sapindus saponaria* (Sapindaceae)	20	30.82	23.77	37.87	100,00	0.000
*Thymus vulgaris* (Lamiaceae)**	1**	402.23	320.98	483.47	**NA**	**NA**
*Xylopia discreta* (Annonaceae)	14	21.85	17.97	25.74	98.60	0.000
*Zanthoxylum schreberi* (Rutaceae)	8	36.44	21.71	51.18	99.90	0.000
*Zingiber officinale* (Zingiberacea)	4	196.03	132.65	259.41	80.70	0.001

Summary of subgroup analyses from 13 studies included in the meta-analysis. Data are presented as pooled mean IC₅₀ values (μg/mL) with 95 % confidence intervals (CI). Subgroups are stratified by type of preparation (crude extracts or derived fractions/compounds), plant part, extraction solvent, *Leishmania* species, and botanical species. Heterogeneity is reported as *I*^*2*^ (%) and corresponding *p* value. “n” indicates the number of records contributing to each estimate. Heterogeneity statistics are not applicable (NA) for subgroups represented by a single record. (**) Subgroups including fewer than three records were considered exploratory, and their pooled estimates should be interpreted with caution due to limited precision and unreliable heterogeneity measures.

At the species level, *Sapindus saponaria* was the most frequently evaluated (n = 20), followed by *Xylopia discreta* (n = 14) and *Ocotea macrophylla* (*n* = 10) (Table 2). Leaves were the most commonly used plant part (*n* = 69), followed by fruits (*n* = 22) and bark/wood (*n* = 21) (Table 2). Among extraction solvents, ethanol was the predominant choice (*n* = 71). The main characteristics of the plant-derived preparations included in this review are summarized in Table 1, Table 2.

### 3.4 IC_50_ and selectivity index values

It is important to note that a low IC₅₀ indicates biological activity but does not necessarily imply selectivity. The selectivity index (SI), defined as the ratio between the cytotoxic concentration to host cells and the inhibitory concentration against *Leishmania spp.*, is a key parameter for assessing how selectively a compound targets the parasite without harming host cells. Unfortunately, a meta-analysis or comparative evaluation based on SI was not feasible in this review, as 26 of the 120 records did not report SI values, and among those that did, only a few provided associated measures of variability.

According to the literature, compounds with SI ≥ 10 are generally considered to exhibit promising antiparasitic activity with acceptable safety margins, whereas values above 20 indicate high potential for further investigation (Peña-Morán et al., 2016; Indrayanto et al., 2021). However, these thresholds should be considered indicative when applied to crude extracts and other types of plant-derived preparations, as they do not reflect true molecular selectivity, which can only be rigorously assessed for isolated compounds. Nevertheless, all 13 studies included in this meta-analysis reported SI values for crude extracts, essential oils, fractions, and/or purified compounds. Accordingly, SI values are presented here as reported by the original authors, in line with their conventions.

For *L*. *braziliensis* promastigotes, the plant-derived preparations exhibiting both the lowest IC₅₀ and highest SI values (≤10 μg/mL and ≥ 20, respectively) were the thymol-chemotype essential oil (IC₅₀ = 0.39 μg/mL; SI = 134.7) and the carvacrol-chemotype essential oil (IC₅₀ = 0.57 μg/mL; SI = 76.7), both extracted directly from *Lippia origanoides* leaves. Likewise, for *L*. *panamensis* promastigotes, the most active preparations were the essential oil and the crude ethanolic extract of *Xylopia discreta* leaves, with IC₅₀ values of 6.35 μg/mL (SI = 110.0) and 9.23 μg/mL (SI = 64.8), respectively. Notably, *X. discreta* consistently demonstrated high SI values across different preparations, underscoring the reliability of its leishmanicidal potential. This consistency supports *X. discreta* as a particularly promising candidate for anti-*Leishmania* drug development (Table 1).

On the other hand, the ethyl acetate leaf extract of *Moquilea salicifolia* demonstrated promising activity against intracellular *L*. *panamensis* amastigotes (IC₅₀ = 9.8 μg/mL; SI > 20.4). Likewise, a fraction derived from the bark/wood ethanolic extract of *Handroanthus chrysanthus* exhibited activity against intracellular *L*. *braziliensis* amastigotes (IC₅₀ = 11.1 μg/mL; SI = 18.9), approaching values commonly referenced in the literature as indicative of promising antileishmanial potential.

### 3.5 Meta-analysis

**Fig. 3:**
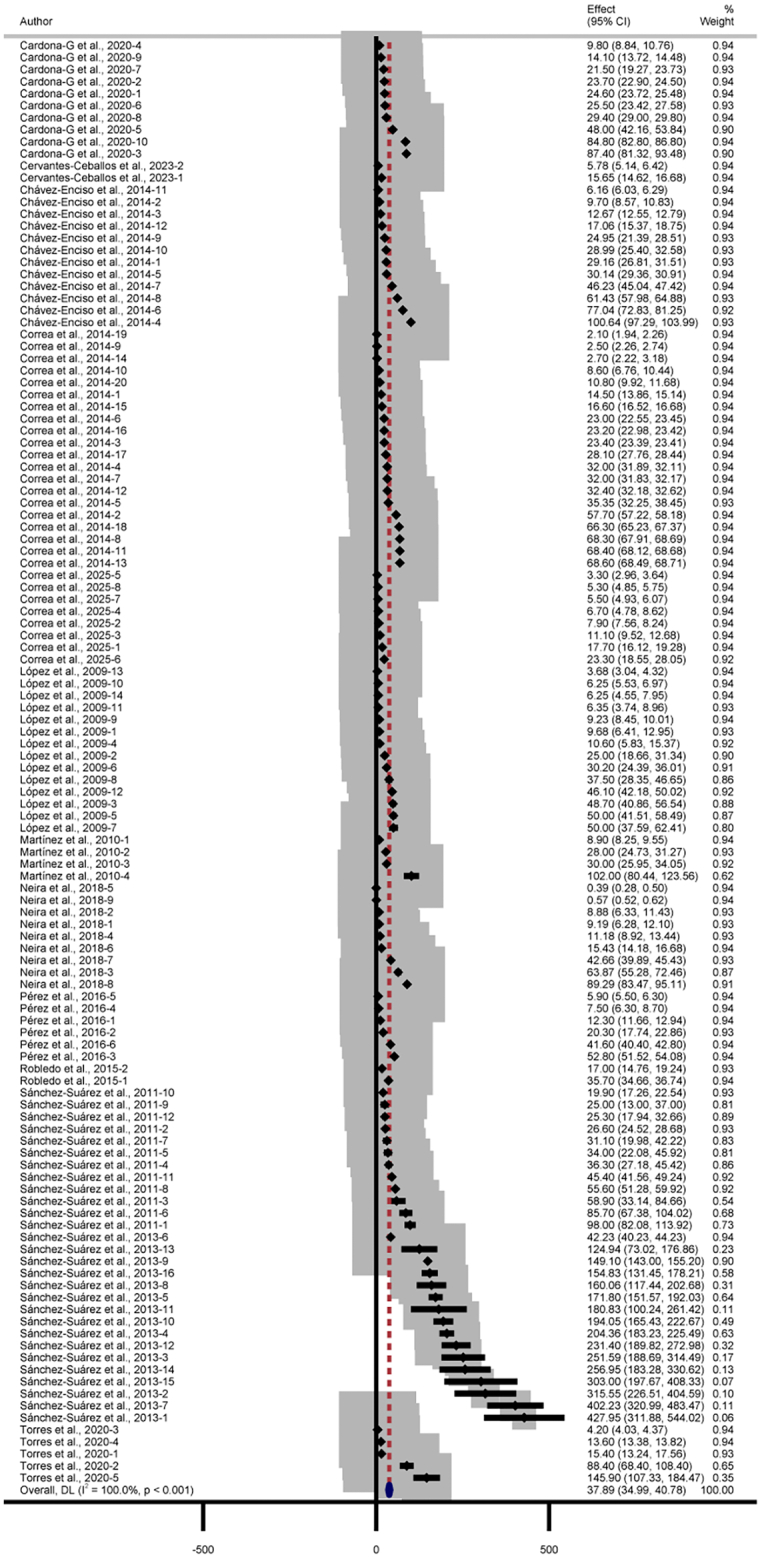
Forest Plot from the Random-Effects Meta-Analysis of the mean IC₅₀ values for Colombian plant-derived preparations with promising antileishmanial activity. The pooled mean IC₅₀ was 37.89 μg/mL (95 % CI: 34.99–40.78). Heterogeneity across studies was assessed using Cochran's Q test (Q = 2.1 × 10^6^, df = 119, *p* = 0.000) and the *I*^*2*^ statistic (*I*^*2*^ = 100.0 %, *p* < 0.001), indicating substantial between-study variability.

Regarding IC₅₀ data, heterogeneity analysis revealed substantial variability among studies (Q = 2.1 × 10^6^, df = 119, *p* = 0.000) (*I*^*2*^ = 100 %, *p* < 0.001), warranting the use of a random-effects model (Fig. 3). The pooled mean IC₅₀ was 37.89 μg/mL (95 % CI: 34.99–40.78). According to commonly used activity thresholds, this value corresponds to a moderate level of *in vitro* antileishmanial activity.

**Fig. 4:**
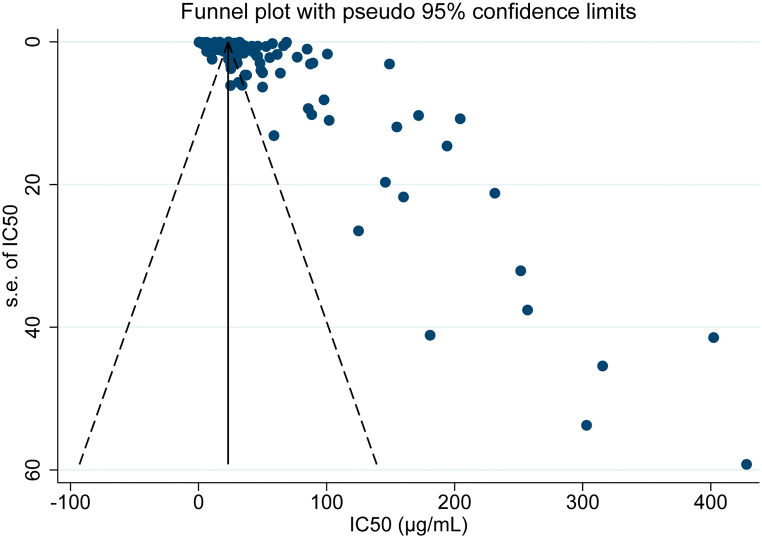
Funnel Plot of studies included in the Meta-Analysis of IC₅₀ values for Colombian plant-derived preparations with promising antileishmanial activity. Funnel plot used to assess publication bias among the studies included in the meta-analysis of IC₅₀ values. Each point represents an individual study, with the x-axis indicating the reported IC₅₀ (μg/mL) and the y-axis representing the standard error of the estimate.

The high heterogeneity (*I*^*2*^ = 100 %) indicates marked variability among studies in terms of plant species, extraction methods, and assay conditions. Visual inspection of the funnel plot (Fig. 4) suggested some asymmetry, with studies concentrated in the upper right quadrant and sparse in the lower right, reflecting uneven data dispersion rather than true publication bias. This pattern likely results from the intrinsic variability among experimental conditions and the underrepresentation of certain categories (*e.g.*, specific plant species, plant part used or type of preparation), rather than genuine publication bias. Consistently, Egger's test did not detect significant publication bias (*p* = 0.519), supporting the robustness of the pooled estimate.

Overall, these results indicate that Colombian plant-derived preparations show a consistent yet moderate level of *in vitro* leishmanicidal activity across studies. However, these findings should be interpreted with caution, as several of the evaluated preparations correspond to crude extracts and other plant-derived preparations, for which this level of activity likely reflects the complexity of plant matrices and the coexistence of bioactive and inactive metabolites.

Additionally, subgroup analyses were conducted according to the plant part used, type of preparation, extraction solvent, *Leishmania* species, and plant species. Subgroups including fewer than three records were considered exploratory, and their pooled estimates should be interpreted with caution due to limited precision and unreliable heterogeneity measures. Most subgroups showed substantial heterogeneity in leishmanicidal activity, with *I*^*2*^ values exceeding 90 % and *p*-values = 0.000 (Cochran's Q test). This high degree of variability can be attributed to pronounced differences in experimental systems across studies, including the plant species analyzed, geographical origin of plant material, plant part evaluated, and preparation methods. Additionally, heterogeneity reflected differences in the *Leishmania* species and strains tested, and in the methodological and experimental conditions of the antileishmanial assays. These findings underscore the critical need to standardize protocols in future studies to facilitate more robust and meaningful comparisons.

Regarding the plant part used, bark/wood and seed preparations showed the lowest mean IC₅₀ values, 22.39 μg/mL (n = 21) and 24.99 μg/mL (n = 4), respectively. The preparations with the lowest mean IC₅₀ values were derived from *Handroanthus chrysanthus* (Bignoniaceae, n = 8), *Moquilea salicifolia* (Chrysobalanaceae, n = 1), *Malachra alceifolia* (Malvaceae, n = 2), *Lippia origanoides* (Verbenaceae, n = 6), and *Xylopia discreta* (Annonaceae, n = 14), with mean IC₅₀ values of 9.36, 9.80, 10.70, 17.71, and 21.85 μg/mL, respectively. Among the solvents used for extraction, methanol, dichloromethane, ethyl acetate, and ethanol yielded the lowest mean IC₅₀ values: 22.98 (n = 2), 24.86 (n = 5), 25.59 (n = 10), and 29.20 μg/mL (n = 71), respectively. Finally, pooled mean IC₅₀ values ranged from 10.70 μg/mL for *L*. *mexicana* (n = 2) to 230.48 μg/mL for *L*. *guyanensis* (n = 4). Detailed data are presented in Table 2.

### 3.6 Ranges-based data and qualitative data

Among the 25 studies included in the analysis, several included IC₅₀ values as ranges rather than exact measurements. For example, some plant-derived preparations were classified as “highly active” (IC₅₀ < 10 μg/mL), “active” (IC₅₀ > 10–50 μg/mL), “moderately active” (IC₅₀ > 50–100 μg/mL), or “inactive” (IC₅₀ > 100 μg/mL), while others studies used similar numerical ranges but different descriptive labels such as “very high” (IC₅₀ < 10 μg/mL), “high” (IC₅₀ > 10–25 μg/mL), “moderate” (IC₅₀ > 25–50 μg/mL), “low” (IC₅₀ > 50–100 μg/mL), or “no” leishmanicidal activity (IC₅₀ > 100 μg/mL). Values reported in this manner are hereafter referred to as range-based data. Although these data could not be incorporated into the quantitative meta-analysis, they still provide valuable insight for identifying potentially promising species, informing research prioritization, and reducing duplication of effort. Nonetheless, enhancing comparability across studies will require the adoption of standardized efficacy metrics alongside appropriate measures of variability.

**Fig. 5:**
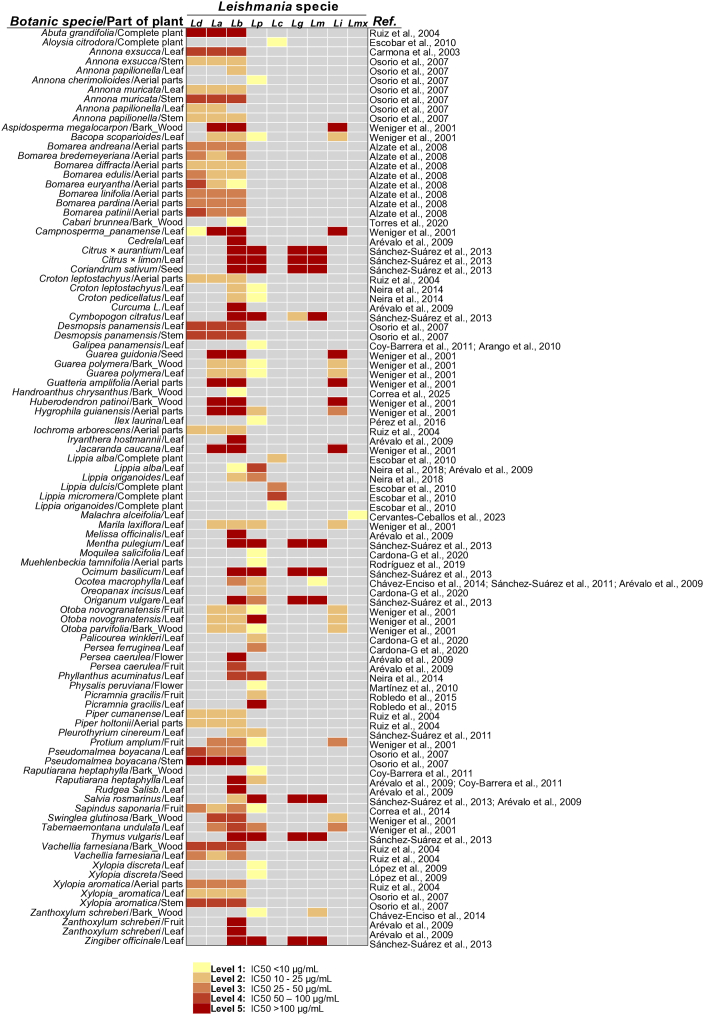
Heat Map of leishmanicidal activity (IC₅₀) for Colombian plant-derived preparations against different *Leishmania* species. Each row represents a combination of botanical species and plant part, showing the lowest IC₅₀ value reported against each *Leishmania* species across the studies included in this review. IC₅₀ values were classified into five categories: <10 μg/mL, 10–25 μg/mL, >25–50 μg/mL, >50–100 μg/mL, and > 100 μg/mL. These thresholds were adapted from Weniger et al. (2001). Gray-shaded cells indicate combinations for which no data were available in the included studies. **Abbreviations: Ld**, *L. donovani*. **La**, *L. amazonensis*. **Lb**, *L. braziliensis*. **Lp**, *L. panamensis*. **Lc**, *L. chagasi*. **Lg**, *L. guyanensis*. **Lm**, *L. major*. **Li**, *L. infantum*. **Lmx**, *L. mexicana.* (Alzate et al., 2008; Arango et al., 2010; Arévalo et al., 2009; Carmona et al., 2003; Coy-Barrera et al., 2011; Escobar et al., 2010; Neira et al., 2014; Osorio et al., 2007; Ruiz et al., 2004)

Quantitative and range-based data are summarized in Fig. 5, which categorizes IC₅₀ values into five intervals: <10 μg/mL, 10–25 μg/mL, >25–50 μg/mL, >50–100 μg/mL, and > 100 μg/mL. The figure displays, for each botanical species and plant part (per *Leishmania* species), the lowest IC₅₀ value reported across the studies reviewed. This visualization facilitates the rapid identification of plant species exhibiting potentially relevant antileishmanial activity across different *Leishmania* species.

Finally, in the study by Calderón et al. (2010), extracts were first evaluated at a fixed concentration of 50 μg/mL against *L*. *mexicana*, and none of the Colombian extracts achieved 50 % inhibition at that concentration. Consequently, IC₅₀ values were reported as >50 μg/mL and the extracts were not further examined by the authors (S4 Table). These results nevertheless provide relevant background, as they document experimental evaluation of those Colombian species and indicate only modest activity under the tested conditions.

## 4 Discussion

The results of this systematic review and meta-analysis demonstrate that several plant species collected in Colombia exhibit promising *in vitro* antileishmanial activity, as reflected by IC₅₀ values comparable to, or in some cases lower than, those reported in previous systematic reviews of plants collected in other countries. The pooled mean IC₅₀ for Colombian plant-derived preparations against *Leishmania* was 37.89 μg/mL (95 % CI: 34.99–40.78), notably lower than the pooled mean reported for plant extracts in Iran, which was 456.64 μg/mL (95 % CI: 396.15–517.12) (Soosaraei et al., 2017). In comparison, the pooled mean IC₅₀ for Ethiopian medicinal plants was 16.80 μg/mL (95 % CI: 12.44–21.16) against promastigotes and 13.81 μg/mL (95 % CI: 13.12–14.50) against amastigotes (Worku et al., 2024).

Although the IC₅₀ is a key parameter for assessing the biological potency of an extract or other plant-derived preparation, it is not sufficient to determine its potential as a pharmacological candidate. *In vitro* efficacy must be evaluated alongside toxicity and selectivity profiles.

A key contribution of this review is the systematic consideration of the SI alongside IC₅₀ values. While IC₅₀ alone reflects antiparasitic potency, it provides limited insight into therapeutic potential in the absence of cytotoxicity data. The SI, by integrating efficacy and host-cell safety, offers a more informative measure of selectivity and risk. Accordingly, plant-derived preparation displaying moderate IC₅₀ values but high SI may represent more promising candidates than highly potent yet cytotoxic preparations. However, caution is necessary when extrapolating SI values from non-purified plant preparations, as their complex composition precludes a precise assessment of molecular selectivity. Consequently, for non-purified mixtures, the SI should be viewed as an indicative rather than absolute measure of safety. When interpreted in conjunction with IC₅₀, it nonetheless provides valuable insight into the balance between efficacy and selectivity, thereby strengthening the pharmacological relevance of the findings synthesized in this review.

In some notable cases, such as *X. discreta*, SI values exceeding 20 (up to 110) were reported, suggesting a highly favorable therapeutic window, particularly for the essential oil tested against *L*. *panamensis* promastigotes. This pattern of leishmanicidal activity combined with low cytotoxicity does not appear to be exclusive to *X. discreta*, as bioactive compounds with similar effects have been reported in other *Xylopia* species. For example, roots of *X. parviflora* collected in Limpopo Province, South Africa, and extracted with dichloromethane inhibited the growth of *L*. *donovani* amastigotes with an IC₅₀ of 5.01 μg/mL and an SI of 10 (Bapela et al., 2017). Likewise, a diterpene glycoside of the ent-kaurene type isolated from the leaves of *X. excellens* collected in Manaus, Brazil, exhibited *in vitro* activity against *L*. *amazonensis* promastigotes (IC₅₀ = 15.23 ± 0.64 μg/mL) (Volpato et al., 2018). Although the authors considered an SI of 1.96 to indicate good selectivity, this interpretation relies on a less conservative threshold and is not supported by cited criteria.

Taken together, these findings reinforce the relevance of the *Xylopia* genus as a promising source of antileishmanial compounds and position the Colombian species *X. discreta*, with its consistently higher SI values, as a particularly compelling candidate for further investigation. These comparisons also underscore the importance of adopting standardized and evidence-based criteria for SI interpretation, which would facilitate comparability across studies and support the identification of truly promising therapeutic candidates.

The study by López et al. (2009) on *X. discreta* illustrates the substantial variability that can occur in IC₅₀ and SI values depending on the extraction solvent. Using the same plant material (leaves), the authors reported IC₅₀ values of 25, 50, and 50 μg/mL for ethanol, ether, and ethyl acetate crude extracts, respectively, with corresponding SI values of >40.00, 3.82, and 2.32 (when U937 cells were used for the cytotoxicity assay) (Table 1). These differences indicate that SI values cannot be compared across extracts without caution, because the SI reflects not only the intrinsic antiparasitic activity but also the chemical variability introduced by different extraction methods.

In this context, solvent choice profoundly influences the metabolite profile of the extracts. Ethanol, particularly at intermediate concentrations (70–80 %), solubilizes a broad spectrum of secondary metabolites with medium to high polarity. Under optimized extraction conditions, such as reflux or ultrasound-assisted extraction, ethanol significantly enhances the recovery of phenolic compounds and flavonoids, which have been widely associated with antioxidant, anti-inflammatory, antimicrobial, and antitumor activities (Phanjaroen et al., 2024; Aibuldinov et al., 2024; Anastasescu et al., 2022). In contrast, highly polar glycosylated metabolites are more efficiently extracted with water, although this non-selective solvent also dissolves hydrophilic impurities that may affect bioactivity measurements. Medium-polarity solvents such as dichloromethane tend to extract bioactive compounds like alkaloids, terpenes, and phenolics, while ether and ethyl acetate show similar but variable affinities depending on compound structure. Methanol extracts, which concentrate moderately polar and partially lipophilic secondary metabolites, have been repeatedly associated with anti-*Leishmania* effects (De Sousa et al., 2020; García-Soriano et al., 2021; Taramelli et al., 2021). It is important to note, however, that methanol is not suitable for pharmaceutical applications due to its toxicity. In research, it is frequently employed as an extraction solvent to profile bioactive compounds, but for further development, safer alternatives such as ethanol or ethyl acetate are typically preferred. Finally, non-polar solvents such as hexane are more likely to extract lipophilic substances like waxes and chlorophylls, which are generally considered non-bioactive in antiprotozoal assays (Azwanida, 2015).

Taken together, these findings illustrate the critical influence of solvent selection on the phytochemical profile and biological activity of plant-derived preparations, and reinforce the importance of rational extraction strategies in antiprotozoal drug discovery.

Another noteworthy finding of this review is the high proportion of studies focused on *L*. *panamensis*, a species with broad geographic distribution and clear clinical relevance in Central America and along the Pacific coast of Colombia and Ecuador. Although this emphasis was expected given the local epidemiology, its formal confirmation is particularly important in a global research context, where other *Leishmania* species are more commonly studied. Previous reviews conducted in regions such as East Africa (*e.g.*, Ethiopia) and Asia (*e.g.*, Iran) have focused on *Leishmania* species that do not reflect Colombian transmission patterns: *L. tropica*, *L. major*, and *L. infantum* in Iran, and *L. aethiopica*, *L. donovani*, and *L*. *major* in Ethiopia. Beyond *X. discreta*, other Colombian plant species exhibited promising selectivity against *L*. *panamensis*, including *Origanum vulgare* (SI = 12.90) against promastigotes, and *Moquilea salicifolia* (SI >20.40), *Picramnia gracilis* (SI >11.80), and *Zanthoxylum schreberi* (SI = 11.60) against amastigotes. In the case of *L*. *braziliensis*, *Handroanthus chrysanthus* exhibited high selectivity (SI = 18.90, amastigotes), while for *L*. *major*, selective activity was observed in *Z. schreberi* (SI = 10.50, amastigotes) and *Ocotea macrophylla* (SI = 10.36, promastigotes).

Collectively, these results indicate that a subset of Colombian plant species possesses selective antileishmanial activity not only against the most prevalent local species but also against others of broader epidemiological relevance. This reinforces their potential value as starting points for drug development beyond the local setting.

A major limitation of this review was the substantial methodological heterogeneity among the included studies. Some of this variability was expected, arising from differences in botanic species, the plant parts used, solvents used for extraction, type of preparation (crude extracts, essential oils, fractions or purified compounds), and the developmental stage of the parasite species targeted (*e.g.*, promastigotes, intracellular amastigotes, or axenic amastigotes). However, additional heterogeneity arose from the lack of standardized protocols, particularly in the methods and criteria used to evaluate antileishmanial activity. This inconsistency hindered meaningful cross-study comparisons and contributed to the high level of statistical heterogeneity observed. Similar challenges have been reported in reviews conducted in other geographical regions, suggesting that this is a broader, structural issue within the field. Therefore, there is a pressing need to establish harmonized experimental guidelines for antileishmanial bioassays. These should include the systematic use of standardized positive controls, generation of complete dose-response curves, and parallel evaluation of parameters such as cytotoxicity in host cells. Adoption of these practices would significantly improve the quality, reproducibility, and translational relevance of the evidence generated.

## 5 Conclusion

This systematic review and meta-analysis provides evidence that several plant species collected in Colombia exhibit promising *in vitro* antileishmanial activity, with IC₅₀ and SI values supporting their potential for further pharmacological development. *Xylopia discreta* emerged as a particularly compelling candidate against *L*. *panamensis*, showing consistently high SI values across multiple extraction methods from leaves and seeds. Similarly, crude leaf extracts of *Moquilea salicifolia* showed high selectivity and strong activity against intracellular *L*. *panamensis* amastigotes, while those of *Lippia origanoides* displayed the highest selectivity and strong inhibitory activity against *L*. *braziliensis*. Importantly, although SI thresholds have been traditionally established for purified compounds and their application to other types of preparations should be approached with caution, this study contributes to a more safety-oriented assessment of plant-derived preparations by integrating SI values. This approach helps to prioritize preparations with more favorable therapeutic windows and guides future efforts toward fractionation, compound isolation, and *in vivo* validation. Despite these promising findings, the high methodological heterogeneity among studies limits the comparability and translational relevance of the available data. Overall, these findings highlight the therapeutic promise of Colombia's flora and underscore its potential as a valuable source of leads for antileishmanial drug discovery.

## CRediT authorship contribution statement

**Carlos Nieto-Clavijo:** Writing – review & editing, Writing – original draft, Visualization, Validation, Methodology, Investigation, Formal analysis, Data curation, Conceptualization. **Liliana Morales:** Writing – review & editing, Writing – original draft, Visualization, Methodology, Investigation, Formal analysis, Conceptualization. **Guillermo Zambrano:** Writing – review & editing, Investigation. **Andrés Delgado-Aldana:** Writing – review & editing, Investigation. **Zayda-Lorena Corredor-Rozo:** Writing – review & editing, Investigation. **Eliana Patricia Calvo:** Writing – review & editing, Investigation. **Dario Tinjacá:** Writing – review & editing, Investigation. **Jacqueline Chaparro-Olaya:** Writing – review & editing, Writing – original draft, Visualization, Supervision, Project administration, Methodology, Investigation, Funding acquisition, Formal analysis, Conceptualization.

## Funding

This project was funded by the Universidad El Bosque (PCI-2023-0031).

## Declaration of competing interest

All authors contributed to the design and conduct of the study and declare that they have no financial interests or personal relationships that could have influenced the reporting of this work.

## Data Availability

All relevant data are within the paper and its Supporting Information files.

## Appendix A Supplementary data

S1 Checklist. PRISMA 2020 checklist Image 1

S1 File. Controlled vocabulary terms and full search strategies Image 2

S1 Table. Study selection process Image 3

S2 Table. Included studies and meta-analysis eligibility Image 4

S3 Table. Risk of bias assessment Image 5

S4 Table. Image 6

S5 Table. Image 6

## References

[bb0005] Aibuldinov Y., Zeinuldina A., Ibrayeva M., Kolpek A., Mukazhanova Z., Nurlybayeva A. (2024). Phytochemical profiling, antioxidant and antimicrobial potentials of ethanol and ethyl acetate extracts of *Chamaenerion latifolium* L. Pharmaceuticals.

[bb0010] Alvar J., Vélez I.D., Bern C., Herrero M., Desjeux P., Cano J. (2012). Leishmaniasis worldwide and global estimates of its incidence. PLoS One.

[bb0015] Alzate F., Jimenez N., Weniger B., Bastida J., Gimenez A. (2008). Antiprotozoal activity of ethanol extracts of some Bomarea species. Pharm. Biol..

[bb0020] Anastasescu M., Badea V., Schröder V., Atkinson I., Musuc A., Popovici V. (2022). Formulation and development of bioadhesive oral films containing Usnea barbata (L.) F.H.Wigg dry ethanol extract with antimicrobial and anticancer properties for potential use in oral cancer complementary therapy. Pharmaceutics.

[bb0025] Arango V., Robledo S., Séon-Méniel B., Figadère B., Cardona W., Sáez J. (2010). Coumarins from Galipea panamensis and their activity against Leishmania panamensis. J. Nat. Prod..

[bb0030] Arévalo Y., Robledo S., Muñoz L., Granados-Falla D., Cuca L.E., Delgado G. (2009). Evaluación in vitro de la actividad de aceites esenciales de plantas colombianas sobre Leishmania braziliensis. Rev. Colomb. Cienc. Quím. Farm..

[bb0035] Azwanida N.N. (2015). A review on the extraction methods use in medicinal plants, principle, strength and limitation. Med. Aromat. Plants..

[bb0040] Bapela M.J., Kaiser M., Meyer J.J.M. (2017). Antileishmanial activity of selected south African plant species. S. Afr. J. Bot..

[bb0045] Calderón A.I., Romero L.I., Ortega-Barría E., Solís P.N., Zacchino S., Gimenez A. (2010). Screening of Latin American plants for antiparasitic activities against malaria, Chagas disease, and leishmaniasis. Pharm. Biol..

[bb0050] Cardona-G W., Robledo S., Alzate F., Yepes A.F., Hernandez C., Velez I.D. (2020). Antileishmanial and cytotoxic activities of four Andean plant extracts from Colombia. Vet. World.

[bb0055] Carmona D., Sáez J., Granados H., Pérez E., Blair S., Angulo A. (2003). Antiprotozoal 6-substituted-5,6-dihydro-α-pyrones from Raimondia cf. monoica. Nat. Prod. Res..

[bib276] Cervantes-Ceballos L., Mercado-Camargo J., Del Olmo-Fernández E., Serrano-García M.L., Robledo S.M., Gómez-Estrada H. (2023). Antileishmanial activity and in silico molecular docking studies of *Malachra alceifolia* Jacq. fractions against *Leishmania mexicana* amastigotes. Trop. med. infect. dis..

[bb0060] Chávez-Enciso N.A., Coy-Barrera E.D., Patiño O.J., Cuca L.E., Delgado G. (2014). Evaluation of the leishmanicidal activity of Rutaceae and Lauraceae ethanol extracts on golden Syrian hamster (*Mesocricetus auratus*) peritoneal macrophages. Indian J. Pharm. Sci..

[bb0065] Correa E., Quiñones W., Robledo S., Carrillo L., Archbold R., Torres F. (2014). Leishmanicidal and trypanocidal activity of *Sapindus saponaria*. Bol. Latinoam. Caribe Plant. Med. Aromat..

[bb0070] Correa E., Robledo S.M., Echeverri F., Quiñones W., Arbeláez N., Murillo J. (2025). In vitro and in vivo leishmanicidal and trypanocidal activities of isoflavans from *Tabebuia chrysantha* (Jacq.) G. Nicholson timber by-products. Exp. Parasitol..

[bb0075] Correa-Cárdenas C.A., Pérez J., Patino L.H. (2020). Distribution, treatment outcome and genetic diversity of Leishmania species in military personnel from Colombia with cutaneous leishmaniasis. BMC Infect. Dis..

[bb0080] Coy-Barrera C.A., Coy-Barrera E.D., Granados-Falla D.S., Delgado-Murcia G., Cuca-Suarez L.E. (2011). Seco-limonoids and quinoline alkaloids from Raputia heptaphylla and their antileishmanial activity. Chem. Pharm. Bull..

[bb0085] De Sousa D., Yepes L., Lopes S., Pérez-Castillo Y., Robledo S. (2020). Alkyl and aryl derivatives based on p-coumaric acid modification and inhibitory action against Leishmania braziliensis and plasmodium falciparum. Molecules.

[bb0090] Escobar P., Leal S.M., Herrera L.V., Martinez J.R., Stashenko E. (2010). Chemical composition and antiprotozoal activities of Colombian Lippia spp essential oils and their major components. Mem. Inst. Oswaldo Cruz.

[bb0095] García-Soriano J., De Lucio H., Vaquero J., Jiménez-Ruiz A., García-Marín J., Sánchez-Alonso P. (2021). Pyridazino-pyrrolo-quinoxalinium salts as highly potent and selective leishmanicidal agents targeting trypanothione reductase. Eur. J. Med. Chem..

[bb0105] Hassan A.A., Khalid H.E., Abdalla A.H., Mukhtar M.M., Osman W.J., Efferth T. (2022). Antileishmanial activities of medicinal herbs and phytochemicals in vitro and in vivo: an update for the years 2015 to 2021. Molecules.

[bb0110] Indrayanto G., Putra G.S., Suhud F. (2021). Validation of in-vitro bioassay methods: application in herbal drug research. Profiles Drug Subst. Excip. Relat. Methodol..

[bb0115] Instituto Nacional de Salud (2024).

[bb0120] Instituto Nacional de Salud (2025).

[bb0125] Koko W.S., Al Nasr I.S., Khan T.A., Schobert R., Biersack B. (2022). An update on natural antileishmanial treatment options from plants, fungi and algae. Chem. Biodivers..

[bb0130] López R., Cuca L.E., Delgado G. (2009). Antileishmanial and immunomodulatory activity of Xylopia discreta. Parasite Immunol..

[bb0135] Majumder N., Banerjee A., Saha S. (2023). A review on new natural and synthetic anti-leishmanial chemotherapeutic agents and current perspective of treatment approaches. Acta Trop..

[bb0140] Martínez W., Ospina L.F., Granados D., Delgado G. (2010). In vitro studies on the relationship between the anti-inflammatory activity of *Physalis peruviana* extracts and the phagocytic process. Immunopharmacol. Immunotoxicol..

[bb0145] Ministerio de Salud y Protección Social (2023).

[bb0150] Neira L.F., Stashenko E., Escobar P. (2014). Actividad antiparasitaria de extractos de plantas colombianas de la familia Euphorbiaceae. Rev. Univ. Ind. Santander Salud..

[bb0155] Neira L.F., Mantilla J.C., Stashenko E., Escobar P. (2018). Toxicidad, genotoxicidad y actividad anti-Leishmania de aceites esenciales obtenidos de cuatro quimiotipos del género Lippia. Bol. Latinoam. Caribe Plant. Med. Aromat..

[bb0160] Osorio E., Arango G.J., Jiménez N., Alzate F., Ruiz G., Gutiérrez D. (2007). Antiprotozoal and cytotoxic activities in vitro of Colombian Annonaceae. J. Ethnopharmacol..

[bb0165] Oualha R., Abdelkrim Y.Z., Guizani I., Harigua-Souiai E. (2024). Approved drugs successfully repurposed against Leishmania based on machine learning predictions. Front. Cell. Infect. Microbiol..

[bib277] Ouzzani M., Hammady H., Fedorowicz Z., Elmagarmid A. (2016). Rayyan-a web and mobile app for systematic reviews. Syst. Rev..

[bb0170] Ovalle-Bracho C., Londoño-Barbosa D., Salgado-Almario J., González C. (2019). Evaluating the spatial distribution of Leishmania parasites in Colombia from clinical samples and human isolates (1999 to 2016). PLoS One.

[bb0175] Page M.J., McKenzie J.E., Bossuyt P.M., Boutron I., Hoffmann T.C., Mulrow C.D. (2021). The PRISMA 2020 statement: an updated guideline for reporting systematic reviews. Syst. Rev..

[bb0180] Pájaro-González Y., Oliveros-Díaz A.F., Cabrera-Barraza J., Cerra-Domínguez J., Díaz-Castillo F., Chassagne F. (2022). Medicinal Plants as Anti-Infectives.

[bb0185] Peña-Morán O.A., Villarreal M.L., Álvarez-Berber L., Meneses-Acosta A., Rodríguez-López V. (2016). Cytotoxicity, post-treatment recovery, and selectivity analysis of naturally occurring Podophyllotoxins from *Bursera fagaroides var. fagaroides* on Breast Cancer Cell Lines. Molecules (Basel, Switzerland).

[bb0190] Pérez J.M., Robledo S., Cardona W., Alzate F., Muñoz D., Herrera A. (2016). Leishmanicidal and cytotoxic activity of extracts and saponins from Ilex laurina (Aquifoliaceae). Trop. J. Pharm. Res..

[bb0195] Phanjaroen T., Boonchalaem T., Haideri M., Chaipoot S., Khiaolaongam W., Laoung-On J. (2024). Effect of different extraction techniques on phenolic profile and phytochemical potential of Gymnema inodorum leaf extract. Molecules.

[bb0200] Robledo S.M., Cardona W., Ligardo K., Henao J., Arbeláez N., Montoya A. (2015). Antileishmanial effect of 5,3′-hydroxy-7,4′-dimethoxyflavanone of Picramnia gracilis Tul. (Picramniaceae) fruit: in vitro and in vivo studies. Adv. Pharmacol. Pharm. Sci..

[bb0205] Ruiz P.G., Garavito G., Acebey C.L., Arteaga L., Pinzon R., Gimenez T.A. (2004). Actividad leishmanicida y tripanocida de algunas plantas reportadas como medicinales en Colombia. Biofarbo.

[bb0210] Sánchez-Suárez J., Coy-Barrera E., Cuca L.E., Delgado G. (2011). Leishmanicidal and cytotoxic activities of extracts and naturally-occurring compounds from two Lauraceae species. Nat. Prod. Commun..

[bb0215] Sánchez-Suárez J., Riveros I., Delgado G. (2013). Evaluation of the leishmanicidal and cytotoxic potential of essential oils derived from ten Colombian plants. Iran. J. Parasitol..

[bb0220] Santos G.A., Sousa J.M., Aguiar A.H.B.M., Torres K.C.S., Coelho A.J.S., Ferreira A.L. (2023). Systematic review of treatment failure and clinical relapses in leishmaniasis from a multifactorial perspective: clinical aspects, factors associated with the parasite and host. Trop. Med. Infect. Dis..

[bb0225] Scheiffer G., Domingues K.Z.A., Gorski D., Cobre A.F., Lazo R.E.L., Borba H.H.L. (2024). In silico approaches supporting drug repurposing for Leishmaniasis: a scoping review. EXCLI J..

[bb0230] Sheth V.H., Shah N.P., Jain R., Bhanushali N., Bhatnagar V. (2024). Development and validation of a risk-of-bias tool for assessing in vitro studies conducted in dentistry: the QUIN. J. Prosthet. Dent..

[bb0235] Soosaraei M., Fakhar M., Hosseini Teshnizi S., Ziaei Hezarjaribi H., Banimostafavi E.S. (2017). Medicinal plants with promising antileishmanial activity in Iran: a systematic review and meta-analysis. Ann. Med. Surg. (Lond.).

[bb0240] Taramelli D., Bassanini I., Basilico N., Ferrandi E., Gabriele E., Sparatore A. (2021). Design, synthesis and in vitro investigation of novel basic celastrol carboxamides as bio-inspired leishmanicidal agents endowed with inhibitory activity against Leishmania Hsp90. Biomolecules.

[bb0245] Torres F., Robledo S.M., Quiñones W., Escobar G., Archbold R., Correa E. (2020). Exploring antiparasitic molecule sources from timber by-product industries—leishmanicidal and trypanocidal compounds from Clathrotropis brunnea Amshoff. Front. Pharmacol..

[bb0250] Torres-Guerrero E., Quintanilla-Cedillo M.R., Ruiz-Esmenjaud J., Arenas R. (2017). Leishmaniasis: a review. F1000Res.

[bb0255] Volpato H., Nakamura C.V., Koolen H.H., de Souza A.D.L., Pinheiro M.L.B. (2018). Antileishmanial activity of a new ent-kaurene diterpene glucoside isolated from leaves of Xylopia excellens RE Fr.(annonaceae). Rec. Nat. Prod..

[bb0260] Weniger B., Robledo S., Arango G.J., Deharo E., Aragón R., Muñoz V. (2001). Antiprotozoal activities of Colombian plants. J. Ethnopharmacol..

[bb0270] Worku K.M., Asfaw B.G., Mamo D.N., Haile Y., Tesfa H., Aemero M. (2024). Medicinal plants with promising antileishmanial activity in Ethiopia: a systematic review and meta-analysis. Medicine (Baltimore).

[bb0275] World Health Organization (2023). https://www.who.int/news-room/fact-sheets/detail/leishmaniasis.

