# Supplementary material for: Plants with promising antileishmanial activity in Colombia: A systematic review and meta-analysis
Source: Parasite Epidemiol Control. 2025 Dec 1;32:e00467. doi: 10.1016/j.parepi.2025.e00467 (PMC12800360; doi:10.1016/j.parepi.2025.e00467)
Supplement: Supplementary file 2 — S1 File. Controlled vocabulary terms and full search strategies [file mmc2.pdf]

S1 File. Controlled vocabulary terms and full search strategies used in PubMed/MEDLINE, Embase, and LILACS.

The table compares controlled vocabulary terms used in the biomedical databases: PubMed/MEDLINE, Embase, and LILACS, according to their respective thesauri. (1) *Medical Subject Headings* (**MeSH**) for PubMed/MEDLINE. (2) ***Emtree Thesaurus*** for Embase. (3) *Health Sciences Descriptors* (**DeCS**) for LILACS, with entries provided in English, Spanish, and Portuguese. The table highlights the presence or absence of standardized terms, as well as term redirects and language-specific equivalents, to inform the construction of comprehensive, multilingual search strategies.

**PubMed/MEDLINE.**	**EMBASE**		**Lilacs**	
**Mesh (1)**	**Emtree Thesaurus (2)**	**DeCS_English (3)**	**DeCS_Spanish (3)**	**DeCS_Portuguese (3)**
Bioprospecting	Bioprospecting	Bioprospecting	Bioprospección	Bioprospecção
Ethnopharmacology	Ethnopharmacology	*Not a DeCS Term*	*Not a DeCS Term*	*Not a DeCS Term*
Medicine, Traditional	Traditional medicine	Medicine, Traditional	Medicina Tradicional	Medicina Tradicional
Phytochemicals	Phytochemical	Phytochemicals	Fitoquímicos	Compostos Fitoquímicos
Phytotherapy	Phytotherapy	Phytotherapy	Fitoterapia	Fitoterapia
Plant Extracts	Plant Extract	Plant Extracts	Extractos Vegetales	Extratos Vegetais
Plants, Medicinal	Medicinal plant	Plants, Medicinal	Plantas Medicinales	Plantas Medicinais
Plant Oils	Vegetable oil	Plant Oils	Aceites de Plantas	Óleos de Plantas
Oils, Volatile	Essential oil	Oils, Volatile	Aceites Volátiles	Óleos Voláteis
Colombia	Colombia	Colombia	Colombia	Colômbia
Not a MeSH term	Colombian		*Not a DeCS Term*	
Leishmania	Leishmania		Leishmania	
Leishmania braziliensis	Leishmania braziliensis		Leishmania braziliensi	
Leishmania donovani	Leishmania donovani		Leishmania donovani	
Leishmania guyanensis	Leishmania guyanensis		Leishmania guyanensis	
Leishmania infantum	Leishmania infantum		Leishmania infantum	
Leishmania major	Leishmania major		Leishmania major	
Leishmania mexicana	Leishmania mexicana		Leishmania mexicana	
Redirects to "Leishmania guyanensis"	Leishmania panamensis		*Redirects to "Leishmania guyanensis"*	
Leishmania tropica	Leishmania tropica		Leishmania tropica	
Leishmaniasis	Leishmaniasis		Leishmaniasis	

Pubmed Search:

For most MeSH descriptors, all associated entry terms were included in the search strategy to ensure exhaustive retrieval. However, for *Leishmaniasis*, *Leishmania*, and all listed *Leishmania* species, preliminary testing showed that entry terms did not yield additional results beyond those retrieved by the preferred descriptors. To avoid unnecessary complexity, these entry terms were omitted from the final search string.

(("Bioprospecting" OR "Ethnopharmacology" OR "Medicine, Traditional" OR "Traditional Medicine" OR "Folk Remedies" OR "Folk Remedy" OR "Remedies, Folk" OR "Remedy, Folk" OR "Medicine, Primitive" OR "Primitive Medicine" OR "Medicine, Folk" OR "Folk Medicine" OR "Medicine, Indigenous" OR "Indigenous Medicine" OR "Home Remedies" OR "Home Remedy" OR "Remedies, Home" OR "Remedy, Home" OR "Ethnomedicine" OR "Phytochemicals" OR "Dietary Phytochemical" OR "Phytochemical, Dietary" OR "Plant Bioactive Compound" OR "Bioactive Compound, Plant" OR "Compound, Plant Bioactive" OR "Plant Biologically Active Compound" OR "Dietary Phytochemicals" OR "Phytochemicals, Dietary" OR "Plant Bioactive Compounds" OR "Bioactive Compounds, Plant" OR "Compounds, Plant Bioactive" OR "Plant Biologically Active Compounds" OR "Plant-Derived Chemical" OR "Chemical, Plant-Derived" OR "Plant Derived Chemical" OR "Phytochemical" OR "Phytonutrient" OR "Plant-Derived Chemicals" OR "Chemicals, Plant-Derived" OR "Plant Derived Chemicals" OR "Phytonutrients" OR "Plant-Derived Compounds" OR "Compounds, Plant-Derived" OR "Plant Derived Compounds" OR "Plant-Derived Compound" OR "Compound, Plant-Derived" OR "Plant Derived Compound" OR "Phytotherapy" OR "Herbal Therapy" OR "Herb Therapy" OR "Plant Extracts" OR "Extracts, Plant" OR "Plant Extract" OR "Extract, Plant" OR "Herbal Medicines" OR "Medicines, Herbal" OR "Plants, Medicinal" OR "Medicinal Plant" OR "Plant, Medicinal" OR "Medicinal Plants" OR "Medicinal Herbs" OR "Herb, Medicinal" OR "Medicinal Herb" OR "Herbs, Medicinal" OR "Pharmaceutical Plants" OR "Pharmaceutical Plant" OR "Plant, Pharmaceutical" OR "Plants, Pharmaceutical" OR "Healing Plants" OR "Healing Plant" OR "Plant, Healing" OR "Plants, Healing" OR "Plant Oils" OR "Oils, Plant" OR "Plant Oil" OR "Oil, Plant" OR "Oils, Vegetable" OR "Vegetable Oils" OR "Vegetable Oil" OR "Oil, Vegetable" OR "Oils, Volatile" OR "Volatile Oils" OR "Oil, Essential" OR "Essential Oil" OR "Oils, Essential" OR "Essential Oils" OR "Volatile Oil" OR "Oil, Volatile") **AND** ("Colombia" OR "Colombian") **AND** ("Leishmania" OR "Leishmania guyanensis" OR "Leishmania major" OR "Leishmania infantum" OR "Leishmania tropica" OR "Leishmania mexicana" OR "Leishmania donovani" OR "Leishmania braziliensis" OR "Leishmaniasis"))

Embase Serch:

In Embase, each Emtree term was exploded using the /exp function to capture all narrower terms within the hierarchical structure. In addition, all synonyms were included for each concept. This procedure was applied to all terms except Colombia and Colombian, as their listed synonyms, Columbia and Colombians, are incorrect: the former refers to a different geographic entity, and the latter is not a valid term.

((leishmaniasis/exp OR 'Leishmania infection' OR 'Leishmania infections' OR 'infection by Leishmania' OR 'infection of Leishmania' OR 'leishmanial infection' OR 'leishmanial infections' OR 'leishmaniases' OR 'leishmaniasis' OR 'leishmaniosis') OR (Leishmania/exp OR 'Leishmania') OR ('Leishmania braziliensis'/exp OR 'Leishmania (Viannia) braziliensis' OR 'Leishmania brasiliensis' OR 'Leishmania braziliensis' OR 'Leishmania braziliensis braziliensis' OR 'Leishmania braziliensis pifanoi' OR 'Leishmania viannia') OR ('Leishmania guyanensis'/exp OR 'Leishmania braziliensis guyanensis' OR 'Leishmania guyanensis') OR ('Leishmania mexicana'/exp OR 'Leishmania mexicana')) AND ((bioprospecting/exp OR 'bio-prospecting' OR 'biodiversity prospecting' OR 'bioprospecting') OR (ethnopharmacology/exp OR 'ethnopharmacology') OR ('traditional medicine'/exp OR 'ethnomedicinal practice' OR 'ethnomedicinal use' OR 'ethnomedicine' OR 'folk medicine' OR 'folk remedy' OR 'indigenous medicine' OR 'medicine, traditional' OR 'native healing' OR 'native medicine' OR 'traditional healing' OR 'traditional indigenous medicine' OR 'traditional medicine' OR 'traditional remedy') OR (phytochemical/exp OR 'phytochemical' OR 'phytochemical agent' OR 'phytochemicals' OR 'phytopharmaceutical' OR 'plant-derived chemical' OR 'plant-derived compound') OR (phytotherapy/exp OR 'phytotherapy') OR ('plant extract'/exp OR 'plant extract' OR 'plant extracts') OR ('medicinal plant'/exp OR 'ethnomedical plant' OR 'ethnomedicinal plant' OR 'medical plant' OR 'medicinal plant' OR 'medicinal plants' OR 'phytomedical plant' OR 'phytomedicinal plant' OR 'phytotherapeutic plant' OR 'plants, medicinal') OR ('vegetable oil'/exp OR 'plant oil' OR 'plant oils' OR 'seed oil' OR 'vegetable fat' OR 'vegetable oil') OR ('essential oil'/exp OR 'essential oil' OR 'oil, rapidly evaporating' OR 'oil, rapidly evapourating' OR 'oil, volatile' OR 'oils, volatile' OR 'rapidly evaporating oil' OR 'rapidly evapourating oil' OR 'volatile oil')) AND ((Colombia/exp OR 'Colombia') OR (Colombian/exp OR 'Colombian'))

LILACS Search: In LILACS, the search was conducted using official DeCS (Health Sciences Descriptors) in English, Spanish, and Portuguese. Because many records in LILACS are published in Spanish or Portuguese without English abstracts, inclusion of all three languages was essential to capture regionally relevant literature. Due to character limits in the LILACS search interface, the final search string included only the official DeCS descriptors, omitting their extensive lists of synonyms. However, an initial exhaustive search (incorporating all DeCS synonyms) was performed, confirming that their inclusion did not alter the retrieval output. Thus, the compacted version preserved the same sensitivity and recall as the fully expanded strategy.

(Bioprospecting OR Bioprospección OR Bioprospecção OR "Medicine, Traditional" OR "Medicina Tradicional" OR Phytochemicals OR Fitoquímicos OR "Compostos Fitoquímicos" OR Phytotherapy OR Fitoterapia OR "Plant Extracts" OR "Extractos Vegetales" OR "Extratos Vegetais" OR "Plants, Medicinal" OR "Plantas Medicinales" OR "Plantas Medicinais" OR "Plant Oils" OR "Aceites de Plantas" OR "Óleos de Plantas" OR "Oils, Volatile" OR "Aceites Volátiles" OR "Óleos Voláteis") AND (Leishmaniasis OR Leishmania OR "Leishmania braziliensis" OR "Leishmania donovani" OR "Leishmania guyanensis" OR "Leishmania infantum" OR "Leishmania major" OR "Leishmania mexicana" OR "Leishmania tropica") AND (Colombia OR Colômbia OR Colombian OR Colombiano OR Colombiana)
