# Supplementary material for: Plants with promising antileishmanial activity in Colombia: A systematic review and meta-analysis
Source: Parasite Epidemiol Control. 2025 Dec 1;32:e00467. doi: 10.1016/j.parepi.2025.e00467 (PMC12800360; doi:10.1016/j.parepi.2025.e00467)
Supplement: Supplementary file 3 — S1 Table. Study selection process [file mmc3.pdf]

Table S1. Summary of the screening and selection process of studies included in the systematic review.

The table lists all 85 records screened in Rayyan during the selection process. Each entry includes the reference, publication year, initial decision in Rayyan, final decision after full-text assessment, reason for exclusion (when applicable), and additional comments providing context for the exclusion. The 25 studies ultimately included in the systematic review are shaded for visual distinction.

Reference	Year	Decision on Rayyan	Decision (after full reading)	Reason for exclusión	Comments
Alzate F, Jiménez N, Weniger B, Bastida J, Giménez A. Antiprotozoal activity of ethanol extracts of some Bomarea species. Pharm Biol. **2008**;46(9):575–8. https://doi.org/10.1080/13880200801968904	2008	**Included**	**Included**	N.A.	None
Arango V, Robledo S, Séon-Méniel B, Figadère B, Cardona W, Sáez J, et al. Coumarins from *Galipea panamensis* and their activity against *Leishmania panamensis*. *J Nat Prod.* **2010**;73(5):1012–4. doi:10.1021/np100146y	2010	**Included**	**Included**	N.A	None
Arévalo Y, Robledo S, Muñoz L, Granados-Falla D, Cuca LE, Delgado G. Evaluación in vitro de la actividad de aceites esenciales de plantas colombianas sobre *Leishmania braziliensis*. *Rev Colomb Cienc Quím Farm.* **2009**;38(2):131–41.	2009	**Included**	**Included**	N.A	None
Calderón AI, Romero LI, Ortega-Barría E, Solís PN, Zacchino S, Giménez A, et al. Screening of Latin American plants for antiparasitic activities against malaria, Chagas disease, and leishmaniasis. *Pharm Biol.* **2010**;48(5):545–53. https://doi.org/10.3109/13880200903193344	2010	**Included**	**Included**	N.A	None
Cardona-G W, Robledo S, Alzate F, Yepes AF, Hernández C, Vélez ID, et al. Antileishmanial and cytotoxic activities of four Andean plant extracts from Colombia. *Vet World.* **2020**;13(10):2178–85.	2020	**Included**	**Included**	N.A	None
Carmona D, Sáez J, Granados H, Pérez E, Blair S, Angulo A, et al. Antiprotozoal 6-substituted-5,6-dihydro-α-pyrones from *Raimondia cf. monoica*. *Nat Prod Res.* **2003**;17(4):275–80.	2003	**Included**	**Included**	N.A	None
Cervantes-Ceballos L, Mercado-Camargo J, Del Olmo-Fernández E, Serrano-García ML, Robledo SM, Gómez-Estrada H. Antileishmanial activity and in silico molecular docking studies of *Malachra alceifolia* Jacq. fractions against *Leishmania mexicana* amastigotes. *Trop Med Infect Dis.* **2023**;8(2):115. doi:10.3390/tropicalmed8020115	2023	**Included**	**Included**	N.A	None
Chávez-Enciso NA, Coy-Barrera ED, Patiño OJ, Cuca LE, Delgado G. Evaluation of the leishmanicidal activity of Rutaceae and Lauraceae ethanol extracts on golden Syrian hamster (*Mesocricetus auratus*) peritoneal macrophages. *Indian J Pharm Sci.* **2014**;76(3):188–97.	2014	**Included**	**Included**	N.A	None
Correa E, Quiñones W, Robledo S, Carrillo L, Archbold R, Torres F, et al. Leishmanicidal and trypanocidal activity of *Sapindus saponaria*. *Bol Latinoam Caribe Plantas Med Aromat.* **2014**;13(4):311–23.	2014	**Included**	**Included**	N.A	None

Reference	Year	Decision on Rayyan	Decision (after full reading)	Reason for exclusión	Comments
Correa E, Robledo SM, Echeverri F, Quiñones W, Arbeláez N, Murillo J, et al. In vitro and in vivo leishmanicidal and trypanocidal activities of isoflavans from Tabebuia chrysantha (Jacq.) G. Nicholson timber by-products. *Exp Parasitol.* **2025**;270:108899. https://doi.org/10.1016/j.exppara.2025.108899	2025	**Included**	**Included**	N.A	None
Coy-Barrera CA, Coy-Barrera ED, Granados-Falla DS, Delgado-Murcia G, Cuca-Suárez LE. Seco-limonoids and quinoline alkaloids from Raputia heptaphylla and their antileishmanial activity. *Chem Pharm Bull.* **2011**;59(7):855–9. https://doi.org/10.1248/cpb.59.855	2011	**Included**	**Included**	N.A	None
Escobar P, Leal SM, Herrera LV, Martínez JR, Stashenko E. Chemical composition and antiprotozoal activities of Colombian Lippia spp. essential oils and their major components. Mem Inst Oswaldo Cruz. **2010**;105:184–90.	2010	**Included**	**Included**	N.A	None
López R, Cuca LE, Delgado G. Antileishmanial and immunomodulatory activity of *Xylopia discreta*. *Parasite Immunol.* **2009**;31(10):623–630.	2009	**Included**	**Included**	N.A.	None
Martínez W, Ospina LF, Granados D, Delgado G. *In vitro* studies on the relationship between the anti-inflammatory activity of *Physalis peruviana* extracts and the phagocytic process. *Immunopharmacol Immunotoxicol.* **2010**;32(1):63–73. doi:10.1080/08923970903143957	2010	**Included**	**Included**	N.A.	None
Neira LF, Mantilla JC, Stashenko E, Escobar P. Toxicidad, genotoxicidad y actividad anti-*Leishmania* de aceites esenciales obtenidos de cuatro quimiotipos del género *Lippia*. *Bol Latinoam Caribe Plantas Med Aromat.* **2018**;17(1):68–83.	2018	**Included**	**Included**	N.A.	None
Neira LF, Stashenko E, Escobar P. Actividad antiparasitaria de extractos de plantas colombianas de la familia *Euphorbiaceae*. *Rev Univ Ind Santander Salud.* **2014**;46(1):15–22.	2014	**Included**	**Included**	N.A.	None
Osorio E, Arango GJ, Jiménez N, Alzate F, Ruiz G, Gutiérrez D, et al. Antiprotozoal and cytotoxic activities *in vitro* of Colombian *Annonaceae*. *J Ethnopharmacol.* **2007**;111(3):630–635. doi:10.1016/j.jep.2007.01.015	2007	**Included**	**Included**	N.A.	None
Pérez JM, Robledo S, Cardona W, Alzate F, Muñoz D, Herrera A. Leishmanicidal and cytotoxic activity of extracts and saponins from *Ilex laurina* (Aquifoliaceae). *Trop J Pharm Res.* **2016**;15(5):973–979.	2016	**Included**	**Included**	N.A.	None
Robledo SM, Cardona W, Ligardo K, et al. Antileishmanial effect of 5,3'-hydroxy-7,4'-dimethoxyflavanone of *Picramnia gracilis* Tul. (Picramniaceae) fruit: In vitro and in vivo studies. Adv Pharmacol Sci. **2015**;2015:978379. doi:10.1155/2015/978379	2015	**Included**	**Included**	N.A.	None
Rodríguez OE, Torrenegra RD, Pombo LM. Trypanocidal, anti-leishmanial, and cytotoxic activity of *Muehlenbeckia tamnifolia* (Kunth) Meins (Polygonaceae). Asian J Pharm Clin Res. **2019**;12(7). doi:10.22159/ajpcr.2019.v12i7.33790	2019	**Included**	**Included**	N.A.	None
Ruiz PG, Garavito G, Acebey CL, Arteaga L, Pinzón R, Giménez TA. Actividad leishmanicida y tripanocida de algunas plantas reportadas como medicinales en Colombia. Biofarbo. **2004**;13:27–30.	2004	**Included**	**Included**	N.A.	None
Sánchez-Suárez J, Coy-Barrera E, Cuca LE, Delgado G. Leishmanicidal and cytotoxic activities of extracts and naturally-occurring compounds from two *Lauraceae* species. Nat Prod Commun. **2011**;6(2):231–4.	2011	**Included**	**Included**	N.A.	None

Reference	Year	Decision on Rayyan	Decision (after full reading)	Reason for exclusión	Comments
Sánchez-Suárez J, Riveros I, Delgado G. Evaluation of the leishmanicidal and cytotoxic potential of essential oils derived from ten Colombian plants. Iran J Parasitol. **2013**;8(1):129–36.	2013	**Included**	**Included**	N.A.	None
Torres F, Robledo SM, Quiñones W, Escobar G, Archbold R, Correa E, et al. Exploring antiparasitic molecule sources from timber by-product industries—leishmanicidal and trypanocidal compounds from *Clathrotropis brunnea* Amshoff. Front Pharmacol. **2020**;11:584668. doi:10.3389/fphar.2020.584668	2020	**Included**	**Included**	N.A.	None
Weniger B, Robledo S, Arango GJ, Deharo E, Aragón R, Muñoz V, et al. Antiprotozoal activities of Colombian plants. J Ethnopharmacol. **2001**;78(2–3):193–200. doi:10.1016/S0378-8741(01)00346-4	2001	**Included**	**Included**	N.A.	None
Granados-Falla D, Gómez-Galindo A, Daza A, Coy-Barrera E, Delgado G, Cuca-Suárez LE, et al. Seco-limonoid derived from Raputia heptaphylla promotes the control of cutaneous leishmaniasis in hamsters (Mesocricetus auratus). Parasitology. **2016**;143(3):289–99. doi:10.1017/S0031182015001717	2016	**Included**	Excluded	Incomplete data	EC50 reported as µM, not convertible to µg/mL with confidence
Jaramillo MC, Arango GJ, González MC, Robledo SM, Vélez ID. Cytotoxicity and antileishmanial activity of Annona muricata pericarp. Fitoterapia. **2000**;71(2):183–6. doi:10.1016/S0367-326X(99)00138-0	2000	**Included**	Excluded	Incomplete data	No IC_{50} data reported
Mesa LE, Vasquez D, Lutgen P, et al. *In vitro* and *in vivo* antileishmanial activity of *Artemisia annua* L. leaf powder and its potential usefulness in the treatment of uncomplicated cutaneous leishmaniasis in humans. *Rev Soc Bras Med Trop.* **2017**;50(1):52–60. doi:10.1590/0037-8682-0457-2016	2017	**Included**	Excluded	Non-Colombian plant species	Artemisia annua L. material was collected in Walferdange Luxembourg
Upegui-Zapata YA, Echeverri F, Quiñones W, et al. Mode of action of a formulation containing hydrazones and saponins against *Leishmania* spp.: role in mitochondria, proteases and reinfection process. Int J Parasitol Drugs Drug Resist. **2020**;13:94–106. doi:10.1016/j.ijpddr.2020.06.004	2020	**Included**	Excluded	Not exclusively plant-derived (includes synthetic compounds)	Focuses mainly on hydrazone derivatives combined with Sapindus saponaria extract; not an exclusive study of natural extracts
Barrera-Adame D, Cuca-Suárez LE, Delgado G, Coy-Barrera E. UFLC-ESI-MS-based profiling, cytotoxicity and antileishmanial activity of *Rhodostemonodaphne crenaticupula*. *Planta Med.* **2013**;79(13 Suppl):PA8. doi:10.1055/s-0033-1351912	2013	Excluded	Excluded	Conference abstract or non-peer-reviewed source.	The record corresponds to a conference abstract, not a full research article.
Barrera-Adame DA, Delgado G, Coy-Barrera ED. Antileishmanial and cytotoxic activities of three plants used in Colombian folk medicine. *Planta Med.* **2013**;79(13 Suppl):PA9.	2013	Excluded	Excluded	Conference abstract or non-peer-reviewed source.	The record corresponds to a conference abstract, not a full research article.
Bautista-Gomez MM, Doerfler J, Del Mar Castro M. Barriers to cutaneous leishmaniasis care faced by indigenous communities of rural areas in Colombia: a qualitative study. *BMC Infect Dis.* **2022**;22(1):302. doi:10.1186/s12879-022-07204-w	2022	Excluded	Excluded	Ethnobotanical or sociocultural study	The article analyzes sociocultural, infrastructural, and systemic barriers to the diagnosis and treatment of cutaneous leishmaniasis in Colombia.
Bernal FA, Coy-Barrera E. In-silico analyses of sesquiterpene-related compounds on selected Leishmania enzyme-based targets. *Molecules.* **2014**;19(5):5550–9. https://doi.org/10.3390/molecules19055550	2014	Excluded	Excluded	Conference abstract or non-peer-reviewed source. Computational study	The record corresponds to a conference abstract describing a molecular docking study.
Bernal-Gutiérrez JM, López-Ortiz AF, Murillo-Perea E, Méndez JJ. Flora silvestre medicinal utilizada por los Kofán colombianos en el tratamiento de la leishmaniasis cutánea. *Rev Cubana Plantas Med.* **2014**;19(4):407–20.	2014	Excluded	Excluded	Ethnobotanical or sociocultural study	The study is ethnobotanical and ethnopharmacological, documenting the traditional uses of plants

Reference	Year	Decision on Rayyan	Decision (after full reading)	Reason for exclusión	Comments
Brito S, Crescente O, Fernández A, Coronado A, Rodríguez N. Eficacia de un ácido kaurénico extraído de la planta venezolana *Wedelia trilobata* (Asteraceae) contra *Leishmania (Viannia) braziliensis*. *Biomedica.* **2006**;26 Suppl 1:180–7.	2006	Excluded	Excluded	Non-Colombian plant species	Venezuelan plants
Castaño-Osorio JC, Giraldo-García AM. Antiparasitic phytotherapy: perspectives, scope and current development. *Infectio*. **2019**;23(2):189–204. https://doi.org/10.22354/in.v23i2.777	2019	Excluded	Excluded	Review (not experimental on plant-derived compounds)	Is a narrative review discussing natural products and potential treatments for various protozoan diseases.
Cervantes-Ceballos L, Sánchez-Hoyos J, Sánchez-Hoyos F, Serrano-García ML, Del Olmo-Fernández E, Gómez-Estrada H, et al. An overview of genus Malachra L.: ethnobotany, phytochemistry, and pharmacological activity. Plants (Basel). **2022**;11(21):2808. doi:10.3390/plants11212808	2022	Excluded	Excluded	Review (not experimental on plant-derived compounds)	The paper is a review summarizing information on the genus Malachra without original experimental results on antileishmanial activity.
Correa E, Cardona D, Quiñones W, Robledo S, Carrillo L, Archbold R, et al. Leishmanicidal activity of *Pycnoporus sanguineus*. *Phytother Res.* **2006**;20(6):497–9. doi:10.1002/ptr.1890	2006	Excluded	Excluded	Non–plant-derived study (e.g., fungi, bacteria, or synthetic compounds)	The study evaluated compounds from fungi, not medicinal plants.
Echeverri F, Quiñones W, Escobar G, Robledo S, Torres F. Why do we have so many molecules and biodiversity but so few antiparasite medicines? *Bol Latinoam Caribe Plantas Med Aromat*. **2018**;17(5):414–25.	2018	Excluded	Excluded	Review (not experimental on plant-derived compounds)	It is a review article without experimental data or IC_{50} values for antileishmanial activity.
Erber AC, Arana B, Bennis I, Bustos M, Cruz I, Darce M, et al. An international qualitative study exploring patients' experiences of cutaneous leishmaniasis: study set-up and protocol. BMJ Open. **2018**;8(6):e021372. doi:10.1136/bmjopen-2017-021372	2018	Excluded	Excluded	Public health, epidemiological, or ecological study	The study explored patients' experiences and perspectives on cutaneous leishmaniasis through interviews.
Fernández S, Gayozo E. In silico molecular docking of Moringa oleifera Lam. compounds with the catalytic and FAD-NADPH-binding site of trypanothione reductase from Leishmania infantum. Rev Colomb Cienc Quím Farm. **2023**;52(3):1421–45. http://dx.doi.org/10.15446/rcciquifa.v52n3.110211	2023	Excluded	Excluded	In silico studies, no in vitro antileishmanial tests	Molecular docking of Moringa oleifera compounds with L. infantum trypanothione reductase
Fonseca MMF, Angulo LC, González PDM, Mancilla PNA, Sánchez SJF, Gutiérrez JC, et al. In vitro antiprotozoal activity of extracts from four plant species collected in the east region of Colombia. Vitae. **2011**; Supl. 11th Congreso Colombiano de Fitoquímica.	2011	Excluded	Excluded	Conference abstract or non-peer-reviewed source.	The record corresponds to a conference abstract, not a full research article.
García-Zebadúa JC, Reyes-Chilpa R, Huerta-Reyes M, Castillo-Arellano JI, Santillán-Hernández S, Vázquez-Astudillo B, et al. The tropical tree Calophyllum brasiliense: a botanical, chemical and pharmacological review. Vitae. **2014**;21(2):126–45.	2014	Excluded	Excluded	Review (not experimental on plant-derived compounds)	The paper is a review proposing potential uses of Calophyllum brasiliense; no experimental assays on Leishmania.
Gómez-Estrada H, Mercado-Camargo J, Cervantes-Ceballos L, Jiménez-Villalobos T, Robledo-Restrepo S. In vitro antileishmanial activity of Heliotropium indicum Linn. and Cordia dentata Poir. fractions. J Pharm Pharmacogn Res. **2015**; Supl. 4th Int Symp Pharmacol Nat Prod.	2015	Excluded	Excluded	Conference abstract or non-peer-reviewed source.	The record corresponds to a conference abstract, not a full research article.
González U, Pinart M, Reveiz L, Alvar J, Arana B, Boelaert M, et al. Designing and reporting clinical trials on treatments for cutaneous leishmaniasis. Clin Infect Dis. **2010**;51(4):409–19. doi:10.1086/655134	2010	Excluded	Excluded	Review (not experimental on plant-derived compounds)	The paper is a methodological review of clinical trial design; no antileishmanial assays included.
Guzmán-Vásquez D, Bonifácio LLN, Sales KGDS, Luna RLN, Tuiran LEP, Dantas-Torres F. First record of Leishmania (Viannia) sp. and high prevalence of Anaplasma marginale and Trypanosoma theileri in Zebu cattle from Zenú communities in northern Colombia. Pathogens. **2025**;14(4):382. doi:10.3390/pathogens14040382	2025	Excluded	Excluded	Veterinary epidemiological study.	Diagnosis of Leishmania, Anaplasma, and Trypanosoma in cattle from indigenous communities

Reference	Year	Decision on Rayyan	Decision (after full reading)	Reason for exclusión	Comments
Hata Y, De Mieri M, Ebrahimi SN, Mokoka T, Fouché G, Kaiser M, et al. Identification of two new phenanthrenones and a saponin as antiprotozoal constituents of Drypetes gerrardii. Phytochem Lett. **2014**;10:cxxxiii–l.	2014	Excluded	Excluded	Non-Colombian plant species	The study evaluated plant species from South Africa
Hata Y, Ebrahimi SN, De Mieri M, Raith M, Brun R, Kaiser M, et al. Antitrypanosomal isoflavan quinones from Abrus precatorius. Fitoterapia. **2014**;93:81–7. doi:10.1016/j.fitote.2013.12.015	2014	Excluded	Excluded	Non-Colombian plant species	The study evaluated plant species from South Africa
Hata Y, Raith M, Ebrahimi SN, Mokoka T, Fouché G, Brun R, et al. Antiprotozoal isoflavan quinones from Abrus precatorius ssp. africanus. Planta Med. **2013**;79(6):492–8. doi:10.1055/s-0032-1328298	2013	Excluded	Excluded	Non-Colombian plant species	The study evaluated plant species from South Africa
Hata Y, Julianti T, Mokoka T, Moodley N, Zimmermann S, Adams M, et al. Ethnopharmacological screening of South African medicinal plants and HPLC-based activity profiling for antiprotozoal leads. Rev Fitoterapia. **2010**; Supl. 11th Congress Int Soc Ethnopharmacol (ISE) & 1er Encuentro Hispano-Portugués de Etnobiología (EHPE).	2010	Excluded	Excluded	Conference abstract or non-peer-reviewed source. Non-Colombian plant species.	The record corresponds to a conference abstract describing extracts from South African plants.
Hata Y, Raith M, Ebrahimi SN, Zimmermann S, Mokoka T, Naidoo D, et al. Antiprotozoal isoflavan quinones from Abrus precatorius. Planta Med. **2012**; Supl. 8th Joint Meeting of AFERP, ASP, GA, PSE and SIF. http://dx.doi.org/10.1055/s-0032-1321293	2012	Excluded	Excluded	Conference abstract or non-peer-reviewed source. Non-Colombian plant species.	The record corresponds to a conference abstract describing extracts from South African plants.
Herrera-Acevedo C, de Menezes RPB, de Sousa NF, Scotti L, Scotti MT, Coy-Barrera E. Kaurane-type diterpenoids as potential inhibitors of dihydrofolate reductase-thymidylate synthase in New World Leishmania species. Antibiotics (Basel). **2023**;12(4):663. doi:10.3390/antibiotics12040663	2023	Excluded	Excluded	In silico studies, no in vitro antileishmanial tests	The study focuses on enzyme inhibition on DHFR-TS and computational docking studies
Jaramillo-Ramírez GI, Tacugue MC, Power GM, Buitrago M, Díaz D, Morales C, et al. A qualitative analysis of the perceptions of stakeholders involved in vector control and vector-borne disease research and surveillance in Orinoquia, Colombia. Trop Med Infect Dis. **2024**;9(2):43. doi:10.3390/tropicalmed9020043	2024	Excluded	Excluded	Unrelated study (not aligned with the review objective)	None
Jürgens FM, Behrens M, Humpf HU, Robledo SM, Schmidt TJ. In vitro metabolism of helenalin acetate and 11α,13-dihydrohelenalin acetate: natural sesquiterpene lactones from Arnica. Metabolites. **2022**;12(1):88. doi:10.3390/metabo12010088	2022	Excluded	Excluded	Non-Colombian plant species. No Anti-Leishmania assays.	Arnica montana is not a Colombian plant. There are no experiments on anti-Leishmania activity.
Jürgens FM, Herrmann FC, Robledo SM, Schmidt TJ. Dermal absorption of sesquiterpene lactones from Arnica tincture. Pharmaceutics. **2022**;14(4):742. doi:10.3390/pharmaceutics14040742	2022	Excluded	Excluded	Non-Colombian plant species. Dermatological study.	Arnica montana is not a Colombian species. The study investigates the dermal absorption of compounds from Arnica tincture.
Jürgens FM, Robledo SM, Schmidt TJ. Evaluation of pharmacokinetic and toxicological parameters of *Arnica* tincture after dermal application in vivo. *Pharmaceutics.* **2022**;14(11):2379. doi:10.3390/pharmaceutics14112379	2022	Excluded	Excluded	Non-Colombian plant species. Pharmacokinetic and toxicological study.	Arnica montana is not a Colombian species. The study focuses on dermal absorption, metabolism, and toxicity of Arnica tincture in rats.
Leal Pinto SM, Muehlmann LA, Ojeda LLM, et al. Nanoemulsions with chloroaluminium phthalocyanine and paromomycin for combined photodynamic and antibiotic therapy for cutaneous leishmaniasis. *Infect Chemother.* **2021**;53(2):342–354. doi:10.3947/ic.2021.0010	2021	Excluded	Excluded	Diagnostic or pharmacological evaluation	The study evaluates a synthetic nanoformulation with photodynamic therapy

Reference	Year	Decision on Rayyan	Decision (after full reading)	Reason for exclusión	Comments
Lozano YY, Giraldo SG, Zapata AC, Escobar JE, Sánchez RM. Medicinal plants with antileishmanial activity on parasites responsible for New World cutaneous leishmaniasis: a systematic review 2018–2022. *J Pharm Pharmacogn Res.* **2023**;11(6):975–1001. doi:10.56499/jppres23.1697_11.6.975	2023	Excluded	Excluded	Review (not experimental on plant-derived compounds)	It is a systematic review on medicinal plants with potential leishmanicidal activity. The work includes one Colombian article, but it is based on a non-Colombian plant.
Mejía-Manzano LA, Ortiz-Alcaráz CI, Parra Daza LE, et al. *Saccharomyces cerevisiae* biofactory to produce naringenin using a systems biology approach and a bicistronic vector expression strategy in flavonoid production. *Microbiol Spectr.* **2024**;12(1):e0337423. doi:10.1128/spectrum.03374-23	2024	Excluded	Excluded	Unrelated study (not aligned with the review objective)	None
Mesa LE, Manrique R, Robledo SM, Tabares J, Pineda T, Muskus C. The performance of the recombinase polymerase amplification test for detecting *Leishmania* DNA from skin lesions of patients with clinical or epidemiological suspicion of cutaneous leishmaniasis. *Trans R Soc Trop Med Hyg.* **2021**;115(12):1427–1433. doi:10.1093/trstmh/trab073	2021	Excluded	Excluded	Diagnostic or pharmacological evaluation	The paper assesses a molecular diagnostic method.
Mokoka TA, Xolani PK, Zimmermann S, et al. Antiprotozoal screening of 60 South African plants, and the identification of the antitrypanosomal germacranolides schkuhrin I and II. *Planta Med.* **2013**;79(14):1380–1384. doi:10.1055/s-0033-1350691	2013	Excluded	Excluded	Non-Colombian plant species	The study evaluated plant species from South Africa
Mokoka TA, Zimmermann S, Julianti T, et al. *In vitro* screening of traditional South African malaria remedies against *Trypanosoma brucei rhodesiense*, *Trypanosoma cruzi*, *Leishmania donovani*, and *Plasmodium falciparum*. *Planta Med.* **2011**;77(14):1663–1667. doi:10.1055/s-0030-1270932	2011	Excluded	Excluded	Non-Colombian plant species	The study evaluated plant species from South Africa
Murillo E, Bernal J, López A. Preliminary phytochemical characterization of anti-*Leishmania* plants used by indigenous communities in Putumayo, Colombia. In: *11th Congreso Colombiano de Fitoquímica*. *Vitae.* **2011**.	2011	Excluded	Excluded	Conference abstract or non-peer-reviewed source.	The record corresponds to a conference abstract, not a full research article.
Ordúz-Díaz LL, Barrera-Adame DA, Coy-Barrera ED. 8-O-4'-connected neolignans as antileishmanial agents: an exploration by molecular docking. In: *61st International Congress and Annual Meeting of the Society for Medicinal Plant and Natural Product Research*. *Planta Med.* **2013**. doi:10.1055/s-0033-1351914	2013	Excluded	Excluded	Conference abstract or non-peer-reviewed source. Computational study	The record corresponds to a conference abstract describing a molecular docking study, not an experimental evaluation of antileishmanial activity in medicinal plants.
Ordúz-Díaz LL, Cuca-Suárez LE, Delgado G, Coy-Barrera ED. *Virola* plants from Colombian Amazon: LC-DAD and LC-MS-based chemical profiling and antileishmanial and cytotoxic activities. In: *61st International Congress and Annual Meeting of the Society for Medicinal Plant and Natural Product Research*. *Planta Med.* **2013**. doi:10.1055/s-0033-1351934	2013	Excluded	Excluded	Conference abstract or non-peer-reviewed source.	The record corresponds to a conference abstract, not a full research article.
Patiño LH, Castillo-Castañeda AC, Muñoz M, et al. Development of an amplicon-based next-generation sequencing protocol to identify *Leishmania* species and other trypanosomatids in leishmaniasis endemic areas. *Microbiol Spectr.* **2021**;9(2):e0065221. doi:10.1128/spectrum.00652-21	2021	Excluded	Excluded	Diagnostic or pharmacological evaluation	The paper focuses on an amplicon-based NGS assay for species identification in hosts and vectors.
Pérez CCA, Santafé PGG, Torres TMG, et al. Lipid composition and evaluation of the antioxidant and leishmanicidal activities of the basidiomycete *Ganoderma* sp. *Rev Cubana Plant Med.* **2016**;21(3):318–331.	2016	Excluded	Excluded	Non–plant-derived study (e.g., fungi, bacteria, or synthetic compounds)	The study evaluated compounds from fungi, not medicinal plants.

Reference	Year	Decision on Rayyan	Decision (after full reading)	Reason for exclusión	Comments
Prieto J, Cortes D, Jaimes L, Laurido C, Vinet R, Martínez JL. *Pseudoxandra sclerocarpa* Maas, Colombian medicinal plant: a review. *Bol Latinoam Caribe Plantas Med Aromat.* **2015**;14(4):308–316.	2015	Excluded	Excluded	Review (not experimental on plant-derived compounds)	The article is a narrative review without experimental data on antileishmanial activity.
Quintero-Pertuz H, Veas-Albornoz R, Carrillo I, et al. Trypanocidal effect of alcoholic extract of *Castanedia santamartensis* (Asteraceae) leaves is based on altered mitochondrial function. Biomed Pharmacother. **2022**;148:112761. doi:10.1016/j.biopha.2022.112761	2022	Excluded	Excluded	Studies targeting other parasites (not Leishmania).	The study evaluated Castanedia santamartensis extracts against Trypanosoma cruzi, not Leishmania species.
Robledo SM, Quinones W, Escobar G, Torres F, Archbold R, Velez ID, Echeverri F. In vivo studies of the therapeutic response and toxicity of saponin- and chromane-derivatives mixtures composition against leishmaniasis. *9th Meeting of AFERP, ASP, GA, JSP, PSE and SIF.* Planta Med. 2016. doi:10.1055/s-0036-1596974	2016	Excluded	Excluded	Conference abstract or non-peer-reviewed source.	The record corresponds to a conference abstract, not a full research article.
Robledo SM, Restrepo A, Yepes LM, Fernandez M, Velez ID. Studies in vitro and in vivo of antileishmanial activity and differential cytotoxicity of *Cannabis* spp. *65th Annual Meeting of the Society for Medicinal Plant and Natural Product Research, GA 2017.* Planta Med Int Open. **2017**. doi:10.1055/s-0037-1608170	2017	Excluded	Excluded	Conference abstract or non-peer-reviewed source.	The record corresponds to a conference abstract, not a full research article.
Robledo SM, Upegui YA, Echeverri F, Velez ID. Antileishmanial activity and cytotoxicity in U937 cells of essential oils from aromatic plants commonly used in traditional medicine. *65th Annual Meeting of the Society for Medicinal Plant and Natural Product Research, GA 2017.* Planta Med Int Open. **2017**. doi:10.1055/s-0037-1608099	2017	Excluded	Excluded	Conference abstract or non-peer-reviewed source.	The record corresponds to a conference abstract, not a full research article.
Robledo SM, Murillo J, Arbeláez N, Montoya A, Ospina V, Jürgens FM, Vélez ID, Schmidt TJ. Therapeutic efficacy of *Arnica* in hamsters with cutaneous leishmaniasis caused by *Leishmania braziliensis* and *L. tropica*. Pharmaceuticals (Basel). **2022**;15(7):776. doi:10.3390/ph15070776	2022	Excluded	Excluded	Non-Colombian plant species	Arnica montana is not a Colombian species.
Robledo SM, Vélez ID, Schmidt TJ. *Arnica* tincture cures cutaneous leishmaniasis in golden hamsters. Molecules. **2018**;23(1):150. doi:10.3390/molecules23010150	2018	Excluded	Excluded	Non-Colombian plant species	Arnica montana is not a Colombian species.
Rodríguez-Villamizar LA, Orozco-Vargas LC, Muñoz-Mantilla G. Impacto del Plan de Atención Básica en la prevención de leishmaniasis cutánea en zonas rurales de Santander, Colombia. Rev Salud Pública (Bogotá). **2006**;8(1):116–28.	2006	Excluded	Excluded	Public health, epidemiological, or ecological study	The study focused on public health programs and prevention.
Sánchez-Suárez J, Bernal FA, Coy-Barrera E. Colombian contributions fighting leishmaniasis: A systematic review on antileishmanials combined with chemoinformatics analysis. Molecules. **2020**;25(23):5704. doi:10.3390/molecules25235704	2020	Excluded	Excluded	Review (not experimental on plant-derived compounds)	This is a systematic and computational review of Colombian antileishmanial compounds, mainly synthetic.
Sánchez-Suárez JF, Coy-Barrera ED, Cuca LE, Delgado G. Evaluation of the antileishmanial potential of plant extracts and metabolites from the family Lauraceae. *11th Congreso Colombiano de Fitoquímica.* Vitae. **2011**.	2011	Excluded	Excluded	Conference abstract or non-peer-reviewed source.	The record corresponds to a conference abstract, not a full research article.
Scotto C, Burger P, Michel T, Khodjet El Khil M, Ginouves M, Prevot G, Blanchet D, Delprete PG, Fernandez X. Antifungal activities and chemical composition of the essential oil of *Lippia micromera* (Verbenaceae) cultivated in French Guiana. *9th Meeting of AFERP, ASP, GA, JSP, PSE and SIF.* Planta Med. **2016**. doi:10.1055/s-0036-1596570	2016	Excluded	Excluded	Conference abstract or non-peer-reviewed source.	The record corresponds to a conference abstract, not a full research article.

Reference	Year	Decision on Rayyan	Decision (after full reading)	Reason for exclusión	Comments
Sequeda-Castañeda LG, Célis C, Gutiérrez S, Luengas-Caicedo PE. *Berberis rigidifolia* Kunth (Berberidaceae), Colombian endemic plant. Pharmacologyonline. **2016**;1:134–8.	2016	Excluded	Excluded	Review (not experimental on plant-derived compounds)	Thi is a compilation of taxonomic, ecological, and phytochemical data on Berberis rigidifolia; no antileishmanial assays.
Souza RD, Fontenele MA, de Almeida NR, Pereira de Araújo J, Barros Gomes PR. Antileishmanial activity of essential oils: a review. Rev Colomb Cienc Quím Farm. **2022**;51(1):68–88.	2022	Excluded	Excluded	Review (not experimental on plant-derived compounds)	Review on essential oils, their inhibitory potential and selectivity. No original antileishmanial assays.
Taylor M, Reza K, Rodríguez LD. Plantas con actividad fotosensibilizadora y potencial terapéutico en leishmaniasis cutánea: hipericina, una alternativa prometedora. Infectio. **2013**;17:90–102. doi:10.1016/S0123-9392(13)70168-1	2013	Excluded	Excluded	Review (not experimental on plant-derived compounds)	It is a narrative review proposing hypericin as a low-cost phototherapeutic alternative for leishmaniasis
Valadeau C, Pabon A, Deharo E, et al. Medicinal plants from the Yanesha (Peru): evaluation of the leishmanicidal and antimalarial activity of selected extracts. J Ethnopharmacol. **2009**;123(3):413–22. doi:10.1016/j.jep.2009.03.041	2009	Excluded	Excluded	Non-Colombian plant species	Ethnobotanical survey took place in Peruvian communities
Vivero RJ, Torres-Gutierrez C, Bejarano EE, et al. Study on natural breeding sites of sand flies (Diptera: Phlebotominae) in areas of *Leishmania* transmission in Colombia. Parasit Vectors. **2015**;8:116. doi:10.1186/s13071-015-0711-y	2015	Excluded	Excluded	Public health, epidemiological, or ecological study	The study focused on the ecology and breeding habitats of sand fly vectors.
Wanying L, Okromelidze MT, Ramírez-Coronel AA, et al. The association of in-utero exposure to polycyclic aromatic hydrocarbons and umbilical liver enzymes. Sci Total Environ. **2023**;889:164220. doi:10.1016/j.scitotenv.2023.164220	2023	Excluded	Excluded	Unrelated study (not aligned with the review objective)	None
61st International Congress and Annual Meeting of the Society for Medicinal Plant and Natural Product Research. Planta Med. **2013**.	2013	Excluded	Excluded	Conference abstract or non-peer-reviewed source.	The record corresponds to a conference review, not a full research article.

Reasons for exclusion	References (n)
Conference abstract or non-peer-reviewed source.	16
Non-Colombian plant species	13
Review (not experimental on plant-derived compounds)	11
Diagnostic or pharmacological evaluation	3
Public health, epidemiological, or ecological study	3
Unrelated study (not aligned with the review objective)	3
Ethnobotanical or Sociocultural study	2
In silico studies	2
Incomplete data	2
Non–plant-derived study (e.g., fungi, bacteria, or synthetic compounds)	2
Not exclusively plant-derived (includes synthetic compounds)	1
Studies targeting other parasites (not Leishmania).	1
Veterinary epidemiological study.	1
