# Supplementary material for: Plants with promising antileishmanial activity in Colombia: A systematic review and meta-analysis
Source: Parasite Epidemiol Control. 2025 Dec 1;32:e00467. doi: 10.1016/j.parepi.2025.e00467 (PMC12800360; doi:10.1016/j.parepi.2025.e00467)
Supplement: Supplementary file 4 — S2 Table. Included studies and meta-analysis eligibility [file mmc4.pdf]

Table S2. Summary of the studies included after full-text screening and their eligibility for meta-analysis.

The table presents the 25 studies that met the inclusion criteria after full-text assessment. Each entry includes the full reference, indication of whether the study was included in the meta-analysis, the reason for exclusion from the meta-analysis (when applicable), and the type of data reported. Quantitative data: IC_{50} values expressed as exact numerical estimates. Ranges-based data: In addition to exact IC_{50} values, some studies also reported activity as IC_{50} ranges (e.g., <10, >10–50, >50–100, >100 µg/mL). Qualitative data: One study tested extracts only at 50 µg/mL and none achieved ≥50% inhibition at that concentration. Thus, IC_{50} values could only be inferred as >50 µg/mL, without assigning a specific value or range. The 13 studies included in the meta-analysis are shaded for visual distinction. **N.A.**: Not applicable.

Reference	Included in the meta-analysis	Reason for exclusion	Quantitative data	Ranges-based data	Qualitative data
Cardona-G W, Robledo S, Alzate F, Yepes AF, Hernández C, Vélez ID, et al. Antileishmanial and cytotoxic activities of four Andean plant extracts from Colombia. *Vet World.* **2020**;13(10):2178–85.	**Yes**	N.A.	**Yes**	**Yes**	No
Cervantes-Ceballos L, Mercado-Camargo J, Del Olmo-Fernández E, Serrano-García ML, Robledo SM, Gómez-Estrada H. Antileishmanial activity and in silico molecular docking studies of *Malachra alceifolia* Jacq. fractions against *Leishmania mexicana* amastigotes. *Trop Med Infect Dis.* **2023**;8(2):115. doi:10.3390/tropicalmed8020115	**Yes**	N.A.	**Yes**	No	No
Chávez-Enciso NA, Coy-Barrera ED, Patiño OJ, Cuca LE, Delgado G. Evaluation of the leishmanicidal activity of Rutaceae and Lauraceae ethanol extracts on golden Syrian hamster (*Mesocricetus auratus*) peritoneal macrophages. *Indian J Pharm Sci.* **2014**;76(3):188–97.	**Yes**	N.A.	**Yes**	No	No
Correa E, Quiñones W, Robledo S, Carrillo L, Archbold R, Torres F, et al. Leishmanicidal and trypanocidal activity of *Sapindus saponaria*. *Bol Latinoam Caribe Plantas Med Aromat.* **2014**;13(4):311–23.	**Yes**	N.A.	**Yes**	No	No
Correa E, Robledo SM, Echeverri F, Quiñones W, Arbeláez N, Murillo J, et al. In vitro and in vivo leishmanicidal and trypanocidal activities of isoflavans from Tabebuia chrysantha (Jacq.) G. Nicholson timber by-products. *Exp Parasitol.* **2025**;270:108899. https://doi.org/10.1016/j.exppara.2025.108899	**Yes**	N.A.	**Yes**	No	No
López R, Cuca LE, Delgado G. Antileishmanial and immunomodulatory activity of *Xylopia discreta*. *Parasite Immunol.* **2009**;31(10):623–630.	**Yes**	N.A.	**Yes**	No	No
Martínez W, Ospina LF, Granados D, Delgado G. *In vitro* studies on the relationship between the anti-inflammatory activity of *Physalis peruviana* extracts and the phagocytic process. *Immunopharmacol Immunotoxicol.* **2010**;32(1):63–73. doi:10.1080/08923970903143957	**Yes**	N.A.	**Yes**	**Yes**	No

Reference	Included in the meta-analysis	Reason for exclusion	Quantitative data	Ranges-based data	Qualitative data
Neira LF, Mantilla JC, Stashenko E, Escobar P. Toxicidad, genotoxicidad y actividad anti-*Leishmania* de aceites esenciales obtenidos de cuatro quimiotipos del género *Lippia*. *Bol Latinoam Caribe Plantas Med Aromat.* **2018**;17(1):68–83.	**Yes**	N.A.	**Yes**	**Yes**	No
Pérez JM, Robledo S, Cardona W, Alzate F, Muñoz D, Herrera A. Leishmanicidal and cytotoxic activity of extracts and saponins from *Ilex laurina* (Aquifoliaceae). *Trop J Pharm Res.* **2016**;15(5):973–979.	**Yes**	N.A.	**Yes**	**Yes**	No
Robledo SM, Cardona W, Ligardo K, et al. Antileishmanial effect of 5,3'-hydroxy-7,4'-dimethoxyflavanone of *Picramnia gracilis* Tul. (Picramniaceae) fruit: In vitro and in vivo studies. Adv Pharmacol Sci. **2015**;2015:978379. doi:10.1155/2015/978379	**Yes**	N.A.	**Yes**	**Yes**	No
Sánchez-Suárez J, Coy-Barrera E, Cuca LE, Delgado G. Leishmanicidal and cytotoxic activities of extracts and naturally-occurring compounds from two *Lauraceae* species. Nat Prod Commun. **2011**;6(2):231–4.	**Yes**	N.A.	**Yes**	**Yes**	No
Sánchez-Suárez J, Riveros I, Delgado G. Evaluation of the leishmanicidal and cytotoxic potential of essential oils derived from ten Colombian plants. Iran J Parasitol. **2013**;8(1):129–36.	**Yes**	N.A.	**Yes**	**Yes**	No
Torres F, Robledo SM, Quiñones W, Escobar G, Archbold R, Correa E, et al. Exploring antiparasitic molecule sources from timber by-product industries—leishmanicidal and trypanocidal compounds from *Clathrotropis brunnea* Amshoff. Front Pharmacol. **2020**;11:584668. doi:10.3389/fphar.2020.584668	**Yes**	N.A.	**Yes**	**Yes**	No
Alzate F, Jiménez N, Weniger B, Bastida J, Giménez A. Antiprotozoal activity of ethanol extracts of some Bomarea species. Pharm Biol. **2008**;46(9):575–8. https://doi.org/10.1080/13880200801968904	No	Standard deviation of IC_{50} values not reported	**Yes**	No	No
Arango V, Robledo S, Séon-Méniel B, Figadère B, Cardona W, Sáez J, et al. Coumarins from *Galipea panamensis* and their activity against *Leishmania panamensis*. *J Nat Prod.* **2010**;73(5):1012–4. doi:10.1021/np100146y	No	Standard deviation of IC_{50} values not reported	**Yes**	**Yes**	No
Arévalo Y, Robledo S, Muñoz L, Granados-Falla D, Cuca LE, Delgado G. Evaluación in vitro de la actividad de aceites esenciales de plantas colombianas sobre *Leishmania braziliensis*. *Rev Colomb Cienc Quím Farm.* **2009**;38(2):131–41.	No	Standard deviation of IC_{50} values not reported	No	**Yes**	No
Calderón AI, Romero LI, Ortega-Barría E, Solís PN, Zacchino S, Giménez A, et al. Screening of Latin American plants for antiparasitic activities against malaria, Chagas disease, and leishmaniasis. *Pharm Biol.* **2010**;48(5):545–53. https://doi.org/10.3109/13880200903193344	No	Non-numerical limits (e.g., >50 µg/mL)	No	**Yes**	No
Carmona D, Sáez J, Granados H, Pérez E, Blair S, Angulo A, et al. Antiprotozoal 6-substituted-5,6-dihydro-α-pyrones from *Raimondia cf. monoica*. *Nat Prod Res.* **2003**;17(4):275–80.	No	Standard deviation of IC_{50} values not reported	**Yes**	No	No
Coy-Barrera CA, Coy-Barrera ED, Granados-Falla DS, Delgado-Murcia G, Cuca-Suárez LE. Seco-limonoids and quinoline alkaloids from Raputia heptaphylla and their antileishmanial activity. *Chem Pharm Bull.* **2011**;59(7):855–9. https://doi.org/10.1248/cpb.59.855	No	The number of experimental replicates was not reported	**Yes**	**Yes**	No
Escobar P, Leal SM, Herrera LV, Martínez JR, Stashenko E. Chemical composition and antiprotozoal activities of Colombian Lippia spp. essential oils and their major components. Mem Inst Oswaldo Cruz. **2010**;105:184–90.	No	Standard deviation of IC_{50} values not reported	**Yes**	**Yes**	No

Reference	Included in the meta-analysis	Reason for exclusion	Quantitative data	Ranges-based data	Qualitative data
Neira LF, Stashenko E, Escobar P. Actividad antiparasitaria de extractos de plantas colombianas de la familia *Euphorbiaceae*. *Rev Univ Ind Santander Salud.* **2014**;46(1):15–22.	No	The number of experimental replicates was not reported	**Yes**	**Yes**	No
Osorio E, Arango GJ, Jiménez N, Alzate F, Ruiz G, Gutiérrez D, et al. Antiprotozoal and cytotoxic activities *in vitro* of Colombian *Annonaceae*. *J Ethnopharmacol.* **2007**;111(3):630–635. doi:10.1016/j.jep.2007.01.015	No	Standard deviation of IC_{50} values not reported	**Yes**	**Yes**	No
Rodríguez OE, Torrenegra RD, Pombo LM. Trypanocidal, anti-leishmanial, and cytotoxic activity of *Muehlenbeckia tamnifolia* (Kunth) Meins (Polygonaceae). Asian J Pharm Clin Res. **2019**;12(7). doi:10.22159/ajpcr.2019.v12i7.33790	No	Standard deviation of IC_{50} values not reported	**Yes**	No	No
Ruiz PG, Garavito G, Acebey CL, Arteaga L, Pinzón R, Giménez TA. Actividad leishmanicida y tripanocida de algunas plantas reportadas como medicinales en Colombia. Biofarbo. **2004**;13:27–30.	No	Standard deviation of IC_{50} values not reported	**Yes**	**Yes**	No
Weniger B, Robledo S, Arango GJ, Deharo E, Aragón R, Muñoz V, et al. Antiprotozoal activities of Colombian plants. J Ethnopharmacol. **2001**;78(2–3):193–200. doi:10.1016/S0378-8741(01)00346-4	No	Standard deviation of IC_{50} values not reported	**Yes**	**Yes**	**Yes**
