# Supplementary material for: Plants with promising antileishmanial activity in Colombia: A systematic review and meta-analysis
Source: Parasite Epidemiol Control. 2025 Dec 1;32:e00467. doi: 10.1016/j.parepi.2025.e00467 (PMC12800360; doi:10.1016/j.parepi.2025.e00467)
Supplement: Supplementary file 5 — S3 Table. Risk of bias assessment [file mmc5.pdf]

S3 Table. Risk of bias assessment

For quality assessment, a modified version of the quality assessment tool for *in vitro* Studies (QUIN tool) was applied. Each study was evaluated based on specific methodological criteria, scoring 2 points for clearly and adequately reported elements, 1 point for insufficiently specified ones, and 0 points for those not reported. NA= not applicable. The total score was expressed as a percentage of the maximum possible, and studies were classified into three quality levels (QL): poor (P) (<50%), moderate (M) (50–75%), or high (H) (>75%). The QUIN Tool includes 12 criteria (C):

C1= Clearly stated aims/objectives
C2= Detailed explanation of sample size calculation
C3= Detailed explanation of sampling technique
C4= Details of comparison group
C5 = Detailed explanation of methodology
C6= Operator details
C7= Randomization
C8= Method of measurement of outcome
C9= Outcome assessor details
C10= Blinding
C11= Statistical analysis
C12= Presentation of results

Author	Year	C1	C2	C3	C4	C5	C6	C7	C8	C9	C10	C11	C12	Total	%	QL
Ruiz-Grace et al.	2004	2	NA	2	0	2	NA	NA	2	NA	NA	1	2	11	78.6	H
Arevalo et al.	2009	0	NA	2	1	2	NA	NA	2	NA	NA	2	2	11	78.6	H
Escobar et al.	2010	2	NA	2	2	2	NA	NA	2	NA	NA	2	2	14	100	H
Neira et al.	2014	2	NA	2	2	2	NA	NA	2	NA	NA	0	2	12	85.7	H
Sanchez-Suarez et al.	2013	2	NA	1	2	2	NA	NA	2	NA	NA	2	2	13	92.9	H
Sanchez-Suarez et al.	2011	0	NA	2	2	2	NA	NA	2	NA	NA	2	2	12	85.7	H
Correa et al.	2025	1	NA	2	2	2	NA	NA	2	NA	NA	2	2	13	92.9	H
Arango et al.	2010	1	NA	1	2	2	NA	NA	2	NA	NA	0	1	9	64.3	M
Lopez et al.	2009	2	NA	2	2	2	NA	NA	2	NA	NA	2	2	14	100	H
Martinez et al.	2010	2	NA	1	2	2	NA	NA	2	NA	NA	2	2	13	92.9	H
Osorio et al	2007	2	NA	2	2	2	NA	NA	2	NA	NA	1	2	13	92.9	H
Weniger et al.	2001	1	NA	2	2	2	NA	NA	2	NA	NA	0	2	11	78.6	H
Calderon et al.	2010	2	NA	2	2	2	NA	NA	2	NA	NA	0	2	12	85.7	H
Torres et al.	2020	2	NA	1	2	2	NA	NA	2	NA	NA	1	2	12	85.7	H
Neira et al.	2018	2	NA	2	2	2	NA	NA	2	NA	NA	2	2	14	100	H
Perez et al.	2016	2	NA	2	2	2	NA	NA	2	NA	NA	1	1	12	85.7	H
Robledo et al.	2015	1	NA	1	2	2	NA	NA	1	NA	NA	2	2	11	78.6	H
Correa et al.	2014	2	NA	2	2	2	NA	NA	2	NA	NA	2	2	14	100	H
Coy-Barrera et al.	2011	2	NA	2	1	1	NA	NA	1	NA	NA	1	2	10	71.4	M
Alzate et al.	2008	2	NA	0	2	1	NA	NA	2	NA	NA	1	1	9	64.3	M
Carmona et al.	2003	1	NA	1	2	2	NA	NA	2	NA	NA	0	2	10	71.4	M
Chávez-Enciso et al.	2014	2	NA	2	2	2	NA	NA	2	NA	NA	0	2	12	85.7	H
Cardona et al.	2020	2	NA	2	2	2	NA	NA	2	NA	NA	1	2	13	92.9	H
Rodríguez et al.	2019	2	NA	2	2	2	NA	NA	2	NA	NA	0	2	12	85.7	H
Cervantes-Ceballos et al.	2023	2	NA	2	2	2	NA	NA	2	NA	NA	1	2	13	92.9	H
