# Supplementary material for: Plants with promising antileishmanial activity in Colombia: A systematic review and meta-analysis
Source: Parasite Epidemiol Control. 2025 Dec 1;32:e00467. doi: 10.1016/j.parepi.2025.e00467 (PMC12800360; doi:10.1016/j.parepi.2025.e00467)
Supplement: Supplementary file 6 — S4 Table. [file mmc6.pdf]

**S4 Table. Colombian plant species tested in the study by Calderón et al., 2010.**

This table summarizes the plant extracts collected in Colombia and evaluated for *in vitro* antileishmanial activity in the study by Calderón et al. (2010). Extracts were tested against *Leishmania mexicana*, and results were expressed as $IC_{50}$ (concentration required to inhibit 50% of parasite viability). **Axenic A:** axenic amastigote. All Colombian extracts showed $IC_{50}$ values > 50 µg/mL and were therefore considered inactive. Specific $IC_{50}$ values and selectivity indices were not reported in the original publication.

| Botanic specie | Part of plant | Extract | *Leishmania* specie | Stage | $IC_{50}$ (µg/mL) |
|---|---|---|---|---|---|
| *Annona_muricata* | Leaf | Ethanol | *L. mexicana* | Axenic A | >50 |
| *Bocconia integrifolia* | | | | | |
| *Calea peruviana* | | | | | |
| *Cinchona pubescens* | | | | | |
| *Eirmocephala brachiata* | | | | | |
| *Ilex guayusa* | | | | | |
| *Iochroma arborescens* | | | | | |
| *Miconia buxifolia* | | | | | |
| *Monochaetum myrtoideum* | | | | | |
| *Piper barbatum* | | | | | |
| *Piper umbellatum* | | | | | |
| *Varronia cylindristachya* | | | | | |
| *Calea jamaicensis* | Complete plant | | | | |
| *Chromolaena leivensis* | | | | | |
| *Piper holtonii* | Aerial parts | | | | |
| *Critonia morifolia* | Fruit | | | | |
